# Association of functional *IL16* polymorphisms with cancer and cardiovascular disease: a meta-analysis

**DOI:** 10.18632/oncotarget.27715

**Published:** 2020-09-08

**Authors:** Victor Hugo de Souza, Josiane Bazzo de Alencar, Bruna Tiaki Tiyo, Hugo Vicentin Alves, Evelyn Castillo Lima Vendramini, Ana Maria Sell, Jeane Eliete Laguila Visentainer

**Affiliations:** ^1^Post Graduation Program in Biosciences and Physiopathology, Department of Clinical Analysis and Biomedicine, State University of Maringá, Paraná, Brazil; ^2^Laboratory of Immunogenetics, Basic Health Sciences Department, State University of Maringá, Paraná, Brazil

**Keywords:** cytokines, alleles, polymorphism, single nucleotide, inflammation

## Abstract

Introduction: Interleukin-16 (IL-16) is a chemotactic cytokine that is found to increase in Cancer and cardiovascular diseases (CVD). Single nucleotide polymorphisms (SNPs) in *IL16* were associated with diseases. Thus, we conducted a systematic review and meta-analysis to evaluate possible associations between *IL16* rs4778889, rs11556218, rs4072111, and rs1131445 SNPs and the risk for cancer or CVD.

Materials and Methods: This study was performed according to the PRISMA statement. Medline, Web of Science, and Scopus databases were systematically reviewed, and a meta-analysis was conducted.

Results: The analysis comprised 6386 individuals with cancer and 2415 with CVD. The SNP rs11556218 was significantly associated with an increased risk for cancer in Chinese in different genetic inheritance models. Also, to the best of our knowledge, this is the first meta-analysis to show an association of rs4778889 with an increased risk of gastric cancer and rs11556218 with an increased risk of CVD in Chinese.

Conclusions: Our meta-analysis suggested that the SNPs rs11556218 and rs4778889 of *IL16* were associated with an increased risk for cancer in Chinese and rs11556218 with increased risk for CVD in Chinese, highlighting the need for further studies on the impact of these polymorphisms on cancer treatment and surveillance.

## INTRODUCTION

Cancer is the second leading cause of morbidity and mortality worldwide. GLOBOCAN (Global cancer statistics) estimated for 2018 a total of 18.1 million people with cancer and 9.6 million deaths from this disease [1]. The most frequent types of cancer differ among populations due to lifestyle and socioeconomic differences. According to a 2019 USA estimate, the three most prevalent types of cancer among men were prostate cancer (3.65 million cases), colorectal cancer (776,000), and melanoma (684,000); among women, these were breast cancer (3.86 million), cervical cancer (807,000), and colorectal cancer (768,000) [2]. In China, 4.3 million new cases and approximately 2.8 million cancer deaths were estimated in 2015. Lung, stomach, liver, esophageal, and colorectal cancer are the leading causes of cancer death among Chinese [3]. Cardiovascular disease (CVD) is the leading cause of morbidity and mortality worldwide, with over 422.7 million estimated cases and 17.92 million deaths in 2015 alone. Coronary artery disease is the most prevalent CVD, followed by stroke [4].

Although cancer and CVD are considered distinct entities, they have similarities and interactions among risk factors (e.g., smoking habits, physical activity, dietary habits, obesity, and diabetes mellitus), supporting a shared biology [5]. Although the rate of cancer death has been decreasing over the years, the risk for CVD in cancer survivors is a health problem that should be monitored by physicians, because individuals in long-term cancer treatment may develop CVD, leading to death or a worsening quality of life [6–8]. CVD in these patients is related to disruption in the organization of the cardiac tissues (such as those caused by chemotherapy, radiotherapy, and other drugs) [6, 9] which can result in several heart problems, hypertension, and cerebrovascular accidents, among other complications. Although chronic inflammation is the link in the pathogenesis of cancer and CVD [10, 11], additional factors may be shared among these diseases, including those that cannot be modified by individuals, such as age, gender, ethnicity, and genetic differences related to the immune response [5].

Cytokines are mediators of the immune response, and some, such as interferon-α (IFN-α), interleukin (IL)-2, and granulocyte macrophage colony stimulating factor (GM-GSF), are currently indicated in cancer treatment. Other cytokines, such as IL-12, -15, and -21, are undergoing clinical trials [12]. Cardiovascular immunotherapy is also being studied for therapeutic use. One strategy is to target the immune system’s ability to regulate arterial inflammation, for instance, neutralizing proinflammatory cytokines such as IL-1β to resolve inflammatory atherosclerosis. Maintaining tissue homeostasis and repairing lesions are also possibilities, which can be carried out through the action of cytokines such as IL-4 and IL-13 [13].

Interleukin-16 (IL-16) is a chemotactic cytokine whose gene (*IL16*) is located on chromosome 15q26.3. The receptor of IL-16 is a CD4 molecule initially described as inhibiting the interaction between CD4 and HIV-1 [14, 15]. This cytokine is coded as a precursor molecule called pro-IL-16, which is cleaved by caspase-3, generating a smaller secreted molecule [16]. The caspase-3 cleavage site is located between the PDZ2 and PDZ3 domains of the precursor protein and allows processing of pro-IL-16 into its mature form [17]. Both pro-IL-16 and mature IL-16 are biologically active. In lymphocytes, pro-IL-16 acts as a transcriptional repressor in the cell, regulating cell cycle progression through its N-terminal domain [18]. Meanwhile, mature IL-16 is secreted by cells such as CD8+ T and B lymphocytes and is responsible for the processes of chemotaxis, cell growth, and differentiation (e.g., increases IL-2 receptor expression).

IL-16 participates in malignant cell proliferation and transformation, acting on a variety of cells involved in the immune response, alone or in conjunction with other cytokines [18–21]. IL-16 levels were directly correlated with gastrointestinal tumor progression [22] and multiple myeloma [23, 24]; therefore, these malignant tumors are suggested targets for anti-IL-16 therapies [25]. Other studies also demonstrated increases in tissue expression and serum concentrations of IL-16 in malignant ovarian tumors [26] and cutaneous T-cell lymphoma [20, 27]. In cardiovascular diseases, high IL-16 serum concentrations were associated with asymptomatic carotid plaques and reduced numbers of cardiovascular events after surgery, suggesting a protective profile in atherosclerosis and the risk of cardiovascular events [28–30]. However, increased levels of this cytokine were also associated with cardiac fibrosis and myocardial stiffening [31], production of proinflammatory cytokines such as IL-1β and IL-6 [32], as well as the risk for acute myocardial infarction [33], being a potential target for CVD treatments [34].

Single nucleotide polymorphisms (SNPs) are common genetic variations that can alter protein composition or gene expression and thus modify the functioning of immune response components such as cytokines. These modifications may lead to changes in the immune response, cell cycle regulation and metabolism, and DNA repair associated with cancer and CVD susceptibility [35, 36]. SNPs may be located in gene promoter regions (altering gene expression and epigenetic modifications), exons (affecting transcription and translation), introns (modifying splicing and regulation), as well as 5′ and 3′ untranslated regions (UTRs) (affecting translation through microRNA binding) [36]. When associations of SNPs with diseases are confirmed, this knowledge may offer greater understanding of disease prevention, prediction, prognosis, and treatment. Four polymorphisms commonly studied for *IL16* are rs4778889 (located in the promoter region), rs11556218 (missense mutation), rs4072111 (missense mutation), and rs1131445 (located in the 3′-UTR).

A better understanding of the connections between cancer and CVD is crucial for identifying and preventing short- and long-term problems in the treatment and prevention of these diseases. In the study of the association of SNPs with diseases, a major analysis tool is meta-analysis. Meta-analysis is the statistical analysis of the evidence from different individual studies, with the aim of integrating them, combining and summarizing their results [37]. Its importance is given by reducing, for example, the standard deviation and the confidence interval, making the result more reliable, in addition to enabling the inclusion of future studies that may be published. The meta-analysis may show an effect that, individually, cannot be observed due to lack of statistical power (limited sample size). Meta-analysis also allows a synthesis of contradictory data even if the statistical power is small [38]. Thus, in this study we performed a systematic review and meta-analysis to evaluate and update information on possible correlations between the polymorphisms rs4778889 T>C, rs11556218 T>G, rs4072111 C>T, and rs1131445 T>C of *IL16* and the risk of cancer or CVD.

## RESULTS

This systematic review included a total of 6386 cancer subjects and 7395 controls in 19 studies for different types of cancer, as well as 2415 individuals with CVD and 2317 controls in 7 studies. The studied population consisted of Chinese and Iranian individuals. The results are shown in Table 1. SNP rs11556218 of *IL16* was statistically associated with the risk of cancer in Chinese (T vs. G; Pooled OR = 1.38; 95% CI 1.23–1.56; random model). Specifically, for gastric cancer in Chinese, the SNP rs4778889 was associated with risk factors for disease development (T vs. C; Pooled OR = 1.18; 95% CI 1.03–1.35; Fixed model). The SNP rs11556218 was also associated with the risk of CVD in Chinese (T vs. G; pooled OR = 1.51; 95% CI 1.07–2.14; random model). Forest plots for all statistically significant comparisons are shown in Supplementary Figure 1. For the selected studies, no statistically significant associations were observed with rs1131445 and rs4072111 and the selected disease groups. For the Iranian population, significance was not found between *IL16* polymorphisms and cancer or CVD in this meta-analysis.

**Table 1:** Meta-analysis results for groups of diseases

Comparison^a^	*n*	Association test		Model	Heterogeneity test			*PBE*^b^	Citation
		OR	CI 95%		*τ*^2^	*P_het_*	*I*^2^		
Cancer - rs11556218 T>G (total case/control number = 5022/5779)									
T vs G	14	1.38	(1.23–1.56)	R	0.04	<0.01	71	0.98	[40, 43, 44, 68, 81–85, 87–89, 93, 98]
G/G+T/T vs T/G	14	1.36	(1.19–1.55)	R	0.04	<0.01	62	0.92	
T/T vs T/G+G/G	14	1.49	(1.28–1.72)	R	0.05	<0.01	70	0.88	
T/T+T/G vs G/G	14	1.56	(1.33–1.84)	F	0.04	0.15	29	0.65	
T/T vs T/G	14	1.43	(1.24–1.66)	R	0.05	<0.01	65	0.90	
T/T vs G/G	14	1.77	(1.50–2.10)	F	0.07	0.06	41	0.93	
T/G vs G/G	14	1.29	(1.08 – 1.53)	F	0.00	0.72	0	0.20	
**Gastric cancer - rs4778889 T>C (total case/control number = 1046/1310)**									
T vs C	3	1.18	(1.03–1.35)	F	0.01	0.15	47	0.35	[68, 81, 93]
T/T + T/C vs C/C	3	1.41	(1.02–1.95)	F	0.00	0.72	0	0.65	
T/T vs C/C	3	1.48	(1.06–2.06)	F	0.00	0.61	0	0.87	
**Cardiovascular disease - rs11556218 T>G (total case/control number = 1643/1685)**									
T vs G	5	1.51	(1.07–2.14)	R	0.17	<0.01	90	0.07	[63, 73, 92, 95, 96]
G/G+T/T vs T/G	5	1.87	(1.08–3.23)	R	0.43	<0.01	92	0.23	
T/T vs T/G+G/G	5	2.00	(1.11–3.63)	R	0.52	<0.01	94	0.24	
T/T vs T/G	5	2.00	(1.08–3.71)	R	0.55	<0.01	94	0.24	

*n*: Number of selected studies; R: Random model; F: Fixed Model; OR: Pooled *Odds Ratio*. ^a^for all comparisons *P*-value < 0.05. ^b^PBE: *P*-value for the linear regression test of funnel plot asymmetry (Egger’s test). Only significant results of OR are presented.

To avoid a possible bias caused by studies whose genotype frequency distribution in the control groups was not in HWE (Hardy–Weinberg equilibrium), stratified analyses were performed including only studies that met this criterion (*P* ≥ 0.05), according to the goodness-of-fit test for HWE. These results are shown in Table 2. After these analyses, the *IL16* SNP rs11556218 remain associated with the risk of cancer in Chinese (T vs. G; pooled OR = 1.41, 95% CI 1.26–1.59; random model) in the genetic inheritance models described in Table 2. In addition, a subgroup analysis was carried out to separate individuals with gastric and renal cancer from other types of cancer (see Table 2 and Supplementary Figure 2). There was a significant association of rs11556218 with risk for carcinoma and osteosarcoma in all evaluated inheritance models (T vs. G; pooled OR = 1.53; 95% CI 1.34–1.73; random model), while no association was observed with gastric and renal cancer.

**Table 2:** Meta-analysis results for studies with control groups in the Hardy–Weinberg equilibrium

Comparison^a^	*n*	Association test		Model	Heterogeneity test			*PBE*^b^	Citation
		OR	CI 95%		*τ*^2^	*P_het_*	*I*^2^		
**Cancer - rs11556218 T>G (total case/control number = 4367/5675)**									
T vs G	13	1.41	(1.26–1.59)	R	0.03	<0.01	68	0.99	[40, 43, 44, 68, 81–84, 87–89, 93, 98]
G/G+T/T vs T/G	13	1.41	(1.23–1.61)	R	0.04	<0.01	61	0.68	
T/T vs T/G+G/G	13	1.54	(1.33–1.78)	R	0.05	<0.01	68	0.74	
T/T+T/G vs G/G	13	1.66	(1.39–1.98)	F	0.04	0.21	23	0.41	
T/T vs T/G	13	1.48	(1.28–1.71)	R	0.05	<0.01	64	0.69	
T/T vs G/G	13	1.92	(1.60–2.30)	F	0.05	0.15	29	0.66	
T/G vs G/G	13	1.31	(1.09–1.58)	F	0.00	0.62	0	0.13	
**Cancer – Carcinoma and other types - rs11556218 T>G (total case/control number = 3521/4398)**									
T vs G	10	1.53	(1.34–1.73)	R	0.02	<0.01	62	0.63	[40, 43, 44, 68, 82–84, 88, 89, 98]
G/G+T/T vs T/G	10	1.53	(1.32–1.77)	R	0.03	0.02	54	0.45	
T/T vs T/G+G/G	10	1.70	(1.46–1.97)	R	0.03	0.01	58	0.41	
T/T+T/G vs G/G	10	1.76	(1.44–2.16)	F	0.07	0.10	39	0.17	
T/T vs T/G	10	1.63	(1.40–1.89)	R	0.03	0.02	54	0.41	
T/T vs G/G	10	2.10	(1.71–2.58)	F	0.06	0.12	35	0.06	
T/G vs G/G	10	1.33	(1.08–1.65)	F	0.02	0.29	16	0.39	
**Cancer – Gastric and renal cancer - rs11556218 T>G (total case/control number = 1154/1515)**									
T vs G	4	1.16	(1.02–1.32)	F	0.01	0.20	35	0.77	[68, 81, 85, 87]
**Cardiovascular disease - rs11556218 T>G (total case/control number = 1405/1507)**									
T vs G	4	1.70	(1.25–2.32)	R	0.10	<0.01	85	0.29	[63, 92, 95, 96]
G/G+T/T vs T/G	4	2.03	(1.09–3.78)	R	0.47	<0.01	93	0.33	
T/T vs T/G+G/G	4	2.33	(1.24–4.41)	R	0.49	<0.01	94	0.49	
T/T+T/G vs G/G	4	1.77	(1.24–2.53)	F	0.18	0.10	49	0.09	
T/T vs T/G	4	2.25	(1.12–4.50)	R	0.59	<0.01	94	0.58	
T/T vs G/G	4	2.50	(1.19–5.25)	R	0.50	<0.01	72	0.39	

*n*: Number of selected studies; R: Random model; F: Fixed Model; OR: Pooled *Odds Ratio*. ^a^for all comparisons *P*-value < 0.05. ^b^PBE: *P*-value for the linear regression test of funnel plot asymmetry (Egger’s test). Only significant results of OR are presented.

An association of *IL16* rs11556218 with the risk for CVD in Chinese was also found for different genetic inheritance models (T vs. G; pooled OR = 1.70; 95% CI 1.25–2.32; random model). An analysis of the covariates evaluated by CVD studies was carried out with the aim of finding a possible source of heterogeneity for the obtained results. These assessed covariates are described in Supplementary Table 2. In this sense, it was not possible to identify in this analysis which covariables could partially explain the qualitative heterogeneity observed in this CVD analysis.

The statistical power after HWE stratification results, considering a subtle genetic effect (1.25), showed a slight reduction for the association between rs11556218 and cancer (from 99.9% to 93.0% after stratification for HWE) but a substantial reduction for rs11556218 and CVD (90.7% to 81.0%) and for rs4778889 and gastric cancer (72.0% to 54.3%). Forest plots for all statistically significant comparisons after stratification of studies with controls in HWE are shown in Supplementary Figure 2.

The mean minor allele frequencies (MAF) observed among the control groups of the selected studies were 21.5% for rs4778889 (C allele), 21.3% for rs11556218 (G allele), 21.5% for rs4072111 (T allele), and 32.3% for rs1131445 (C allele). These frequencies were similar to those described in the 1000Genomes database for East Asian populations (21.2%, 16.5%, 20.8%, and 31.2%, respectively), and the Fisher test did not indicate a statistically significant difference between the allele frequencies in these populations (*P* > 0.05).

Egger’s tests did not indicate possible bias in the selection of publications (*P* ≥ 0.05), and these results are shown in Tables 2 and 3. The funnel plot for visual assessment is available in Supplementary Figure 3. It is noteworthy that the bias in funnel plots should be interpreted carefully for a meta-analysis with a small number of studies (around 10 or less), since tests in a small number of studies have limited statistical power due to the great heterogeneity of these datasets [39]. The quality assessment of the studies selected by the NOS (Table 3) indicated a 95% score of the studies as reasonable or good (greater than 5).

**Table 3:** Newcastle–ottawa quality assessment scale for selected studies

Study	Selection				Comparability^a^	Exposure			Total score
Shih *et al.*[43]	^*^	^*^	^*^	^*^	^**^	^*^	^*^		8
Wu *et al.* [44]	^*^	^*^	^*^	^*^	^**^	^*^	^*^		8
He *et al*. [81]	^*^	^*^	^*^	^*^	^**^	^*^	^*^	^*^	9
Li *et al*. [82]	^*^	^*^	^*^	^*^	^**^	^*^	^*^		8
Yang *et al*. [73]	^*^	^*^			^*^	^*^	^*^		5
MaiMaiTiMin *et al*. [83]	^*^	^*^	^*^	^*^	^**^	^*^	^*^		8
Tang *et al*. [84]	^*^	^*^	^*^	^*^	^**^	^*^	^*^		8
Yang *et al*. [85]	^*^	^*^		^*^	^**^	^*^	^*^		7
Yao *et al*. [86]	^*^		^*^	^*^	^**^	^*^	^*^		7
Kashfi *et al*. [67]	^*^			^*^	^**^	^*^	^*^		6
Wang and Zhu [87]	^*^	^*^	^*^	^*^	^**^	^*^	^*^		8
Luo *et al*. [88]	^*^	^*^	^*^	^*^	^**^	^*^	^*^		8
Qin *et al*. [89]	^*^	^*^	^*^	^*^	^**^	^*^	^*^		8
Hai-Feng *et al*. [90]	^*^	^*^	^*^	^*^	^**^	^*^	^*^	^*^	9
Huang *et al*. [91]	^*^	^*^	^*^	^*^	^**^	^*^	^*^		8
Liu *et al*. [63]	^*^	^*^		^*^	^**^	^*^	^*^		7
Tong *et al*. [92]	^*^	^*^	^*^	^*^	^**^	^*^	^*^		8
Zhang and Wang [93]	^*^	^*^	^*^	^*^	^**^	^*^	^*^	^*^	9
Azimzadeh *et al*. [72]	^*^	^*^	^*^	^*^	^**^	^*^	^*^		8
Azimzadeh *et al*. [94]	^*^	^*^	^*^	^*^	^**^	^*^	^*^		8
Chen *et al*. [95]^*^	^*^	^*^	^*^	^*^	^**^	^*^	^*^		8
Li *et al*. [40]	^*^		^*^	^*^	^**^	^*^	^*^		7
Wu *et al*. [96]	^*^		^*^		^**^	^*^	^*^		6
Zhu *et al*. [97]	^*^	^*^	^*^	^*^	^**^	^*^	^*^		8
Gao *et al*. [98]	^*^		^*^	^*^	^*^	^*^	^*^		6
Gao *et al*. [68]	^*^	^*^	^*^	^*^	^**^	^*^	^*^		8

Each category can be awarded with one point (^*^). ^a^Up to two points can be given to this category (^**^) when additional factors are controlled.

## DISCUSSION

This systematic review included a total of 6386 individuals with cancer and 2415 individuals with CVD, which confers high statistical power to verify weaker associations. The *IL16* rs11556218 polymorphism was significantly associated with the risk of cancer in Chinese, in different models of genetic inheritance. In addition, to the best of our knowledge, this is the first meta-analysis to show an association between the rs477889 polymorphism and the risk of gastric cancer and the first to demonstrate an association of rs11556218 and the risk of CVD in Chinese.

The association between *IL16* rs11556218 and cancer or CVD remained even after careful analysis of studies control groups according to HWE criteria, and perhaps the loss of association observed for rs4778889 and gastric cancer after stratification was due to the significant loss of statistical power when the sample size of the combined studies was reduced. We also see different results when different types of cancer are grouped together. In our meta-analysis, the subgroup of renal and gastric cancer was not associated with rs11556218. These different results in distinct subgroups can be explained by SNPs having different behaviors in different types of cancer [40]. In addition, sample size limitations should be considered in analyzes of smaller number of studies. Finally, selection bias should not be ruled out in the analysis of different studies.

**Figure 1:**
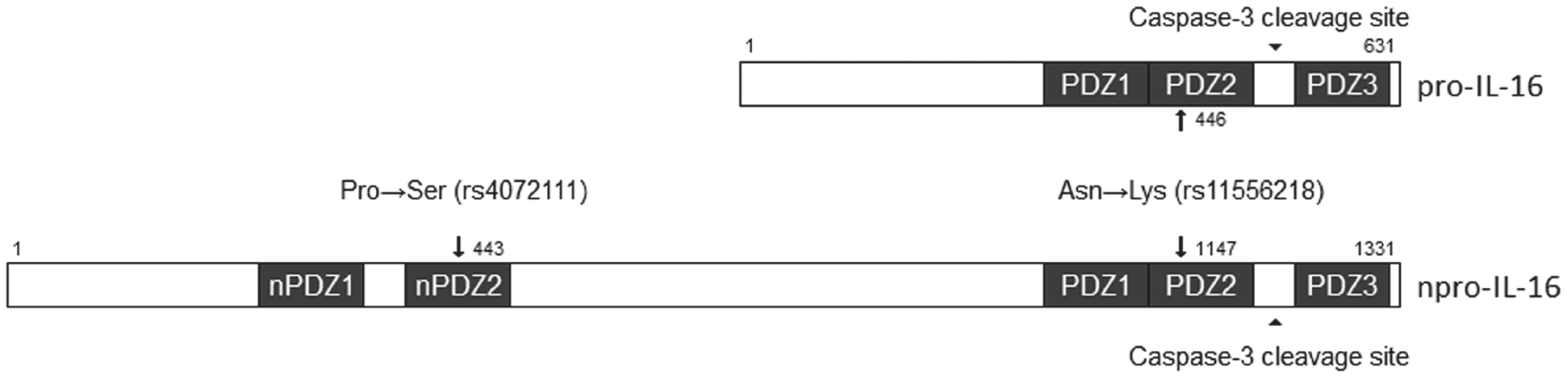
Schematic representation of two SNPs changes in PDZ domains of pro-IL-16 and npro-IL-16. The rs11556218 polymorphism alters the PDZ2 domain at positions 446 and 1147 of pro-IL-16 and n-pro-IL-16, respectively. This polymorphism will cause a substitution of an asparagine to a lysine at the protein, affecting protein recognition by its receptor. The rs4072111 polymorphism is present in the nPDZ2 domain of npro-IL-16 and will cause a substitution of a proline to serine in the protein precursor. Adapted from Bannert *et al.* [17].

In agreement with our results, two other meta-analyses published in 2014 did not find a significant association between rs4778889 and rs4072111 of *IL16* and cancer. However, these same studies observed a statistically significant association between rs11556218 and cancer, an association observed in our study with the addition of new studies published after these meta-analyses results [41–44]. The SNP rs11556218, consisting of a substitution of the T nucleotide by a G, is located in the exon region of the gene and results in a modification of the pro-IL-16 PDZ2 domain (IL-16 isoform with 631 amino acids) and npro-IL-16 (neuronal IL-16 isoform with 1331 amino acids). This modification consists of a substitution of asparagine for lysine in the protein at positions 446 (pro-IL-16) and 1147 (npro-IL-16), as illustrated in Figure 1. This mutation may result in structural changes in the protein, consequently affecting its function in the immune response [45]. This SNP was also associated with prostate cancer in a replication GWAS study in African Americans [46]. It has also been associated with asparaginase-related thrombosis and pancreatitis in the treatment of acute lymphoid leukemia in Caucasians [47], diabetes mellitus [48], autoimmune diseases [49, 50], endometriosis [45, 51], and knee osteoarthritis [52, 53].

A meta-analysis published in 2019 focusing only on renal cancer association studies also found no association between rs4778889 and rs11556218 and this disease [54]. A possible explanation for this lack of association may be related to the limited statistical power to verify subtle associations, due to the small number of individuals in the evaluated studies (total of 790 individuals). Another meta-analysis, published in 2014, found no association between rs4778889 and cancer, which was confirmed in our study for the general cancer group [55]. However, we observed, for the first time, a statistically significant association of this polymorphism with gastric cancer in Chinese. This SNP in the promoter region of the *IL16* gene was related to changes in expression [56]. It was previously associated with precancerous gastric lesions [57], gestational diabetes [58], autoimmune diseases [50, 59], knee osteoarthritis [53], endometriosis [60], Crohn’s disease [61], and allergic contact dermatitis [62].

The SNP rs4072111, consisting of a substitution of the C nucleotide by a T, is located in an intronic region and results in a modification of the nPro-IL-16 PDZ2 domain, with a serine to proline exchange at position 434 [45]. In our study, we found no association of this polymorphism with cancer. Moreover, it was not possible to perform a meta-analysis of the association between rs4072111 and CVD due to the limited number of studies, and no association was observed in the study of Liu *et al*. [63]. This SNP was previously identified as a marker candidate for aggressive prostate cancer [64], although this was not confirmed for an African American population [46, 65]. This polymorphism was also correlated with better prognosis for chronic lymphoid leukemia in an English population [66], associated with the risk of liver cancer [40], colorectal and gastric cancer [67, 68], autoimmunity [49, 50], endometriosis [51], and knee osteoarthritis [52, 53].

The polymorphism rs1131445 (T>C) is located in the 3′-UTR of the gene, and the mutation predicts disruption of the binding of a microRNA inhibitor (miR135b-mRNA) of the gene sequence, which would result in greater pro-IL-16 expression [69]. In our study, we did not observe any association of this SNP with cancer or CVD. Studies have found that this SNP has been associated with cervical cancer in Chinese and prostate cancer in African Americans [46, 70]. Its mutant allele may also be informative about the time to prostate cancer diagnosis among African Americans [71]. It was also associated with gastric and colorectal cancer in Iranians [67, 72], CVD in Chinese [73], Graves’ disease in Chinese [59], and endometriosis in Iranian women [51].

Important limitations should be considered in our study. First, every systematic review presents some risk of bias, related to the difficulty of obtaining non-indexed studies, and the heterogeneity present in the selected studies. Moreover, the results obtained are limited to the populations surveyed and, therefore, lack information for a meta-analysis on the frequency distributions of these polymorphisms in other populations, although an attempt to broadly describe the literature has been made. Similarly, important covariates must be considered to explain the associations observed in these studies, thus multivariate methods of analysis can be useful, highlighting the need for detailed publication of the profile of the populations studied, although the reliability of these methods depends on 10 or more studies. From the results of this review, further studies may be necessary to clarify the role of these polymorphisms in the immunopathogenesis of cancer and CVD, also helping to clarify the relationship between these diseases.

The SNP rs11556218 of *IL16* was significantly associated with an increased risk of cancer or CVD in Chinese. Additionally, the SNP rs4778889 was associated with an increased risk of gastric cancer, while the rs4072111 and rs1131445 SNPs were not associated with cancer or CVD in our study.

## MATERIALS AND METHODS

**Figure 2:**
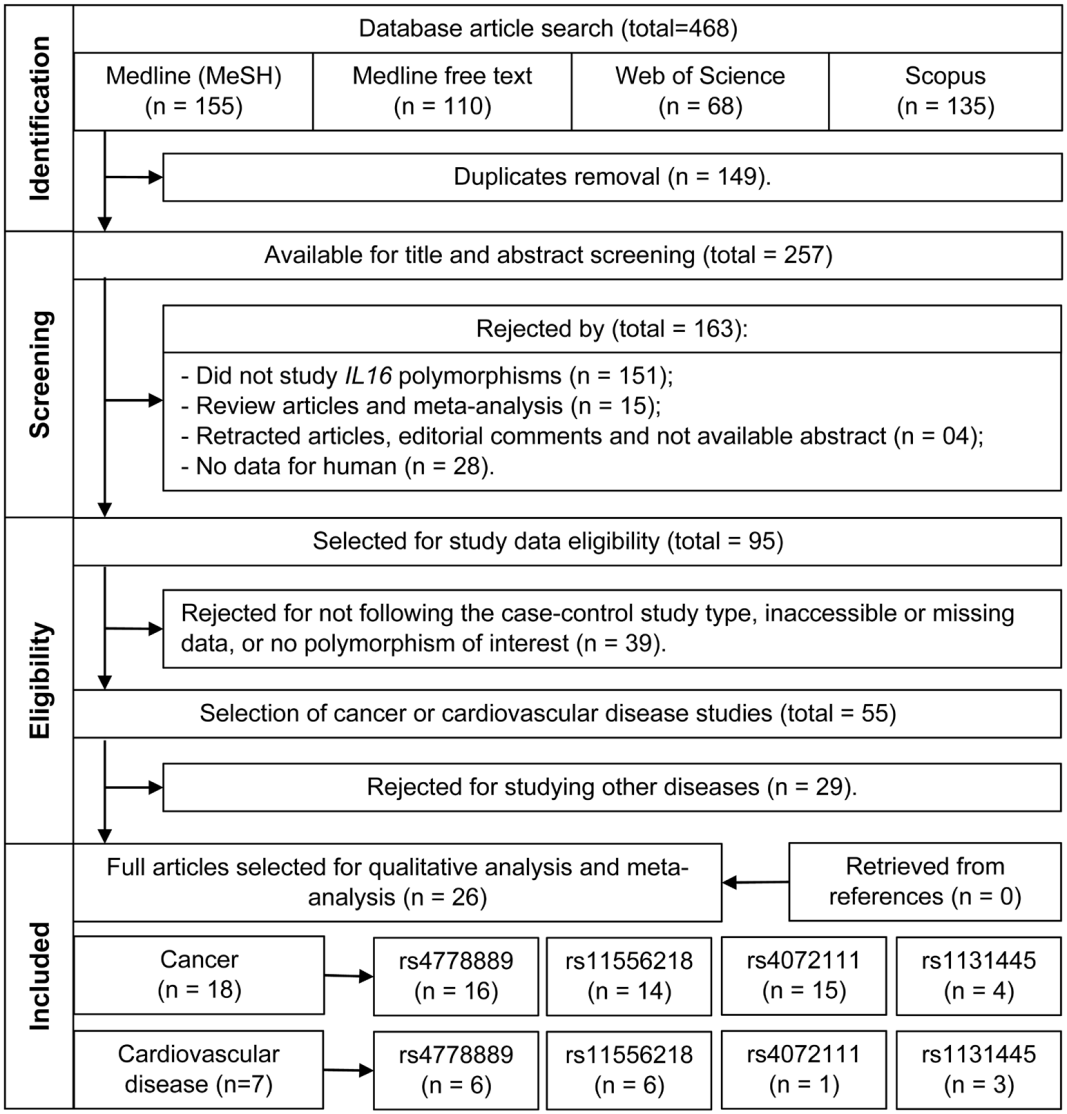
Flowchart of the studies selection.

The methodology of this study was performed according to the PRISMA (Preferred Reporting Items for Systematic Reviews and Meta-Analyses) statement [74]. To identify studies investigating associations between *IL16* polymorphisms and cancer or CVD, the MEDLINE, Web of Science and Scopus databases were searched up to June 30, 2020, according to the strategy detailed in Supplementary Table 1. The language of publication was not defined as a search criterion, but only articles in the English language were included. All steps were performed by two pairs of reviewers in duplicate. Article screening procedures are included in Figure 2.

Articles with available abstracts were selected for title and abstract screening as to their relevance and fitness to the proposed theme. Only original human studies of *IL16* polymorphisms were included. In the next step, the 95 selected articles were evaluated by reading the full text, as to their suitability for inclusion in a meta-analysis (case-control study, evaluating at least one of the polymorphisms of interest, availability of genotype frequency data). Thus, 55 articles were obtained that met all these criteria. All doubts regarding the eligibility of a study or not (according to the criteria described above) were discussed among all authors and resolved after consensus. After a consensus was reached between two authors working in pairs, 26 studies were selected for data collection, according to the diseases of interest. Also, the references of these studies were searched for additional studies. The studies and SNPs selected in this review are described in Supplementary Table 3. The 26 selected studies were further reassessed by a third independent pair of authors, composed of authors with more experience in the field of cytokine polymorphisms and association studies, evaluating whether all the studies and results were in accordance with the proposed criteria for this review.

Genotype frequency data were collected from the selected studies. Allele frequencies were obtained by counting the total number of alleles in all genotypes, and the number of individuals used in the meta-analysis was obtained by summing the total number of described genotypes. Distinct groups of cases or controls within the same study were combined into a single group for distinct and unrelated groups of individuals. The cancer group was analyzed by combining all cancer types or individually for each type of cancer (when a comparison was possible). Statistical analyses were performed by using the program R, version 3.5.2, with the meta-analysis package “meta” [75].

The meta-analysis results are represented by the Forest plot. This chart aims to summarize the results of the pooled studies giving them a general trend. It represents the studies with the proportions of events of interest in each study for the case-control groups. The percentage values of weight and effect size (represented by the *Odds Ratio*, OR) of each study in the final result are presented in two possible models according to observed heterogeneity: fixed and random. Thus, for all comparisons performed, Forest plots were constructed with a heterogeneity test (I^2^). When the value of I^2^ was greater than 50% and the test showed statistically significant results, the random OR model was chosen. When I^2^ was less than 50% with no evidence of significant heterogeneity, the fixed model was chosen. Six comparison groups of alleles and genotypes were used to verify the influence of genetic association with diseases. Considering “M” as the wild-type allele and “m” as the mutated allele, the combinations analyzed were (1) M vs. m alleles; (2) MM vs. Mm genotypes; (3) MM vs. mm and (4) Mm vs. mm; and also genetic inheritance models (5) MM + Mm vs. mm (recessive); (6) MM vs. Mm + mm (dominant); and (7) MM + mm vs. Mm (overdominant).

Quality evaluation of the selected studies was performed according to the Newcastle–Ottawa (NOS) scale by two independent authors. Disagreements were resolved by consensus, and studies with a score higher than 5 were considered as good or reasonable [76]. Possible biases of published meta-analyses were verified by Egger’s test analysis [77], funnel plot visual evaluation, and Begg’s test [78]. All individual study results were expressed as ORs with a 95% confidence interval (CI). The Hardy–Weinberg equilibrium (HWE) of the control group of each study was checked according to the chi-square test for goodness-of-fit (*P* < 0.05), as recommended for assessing study quality [79]. After this, statistical power analysis was performed using the QUANTO software [80], to evaluate sample size effects on the statistical power. The reference population allele frequencies used for comparison were obtained from the 1000 Genomes Project (phase3 release V3+, available at: https://www.internationalgenome.org/) and the comparison was made using the exact Fisher test in R. For all tests, *P* < 0.05 was considered to indicate statistical significance.

## SUPPLEMENTARY MATERIALS





## Abbreviations

GLOBOCAN: Global cancer statistics
CVD: Cardiovascular disease
IFN-α: Interferon-α
IL: Interleukin
GM-GSF: Granulocyte macrophage colony stimulating factor
SNP: Single nucleotide polymorphism
OR: *Odds Ratio*
CI: Confidence interval
HWE: Hardy–Weinberg equilibrium
NOS: Newcastle–Ottawa Scale
GWAS: Genome-wide association study
PRISMA: Preferred Reporting Items for Systematic Reviews and Meta-Analyses
