# Supplementary material for: Association of functional IL16 polymorphisms with cancer and cardiovascular disease: a meta-analysis
Source: Oncotarget. 2020 Sep 8;11(36):3405–17. doi: 10.18632/oncotarget.27715 (PMC7486693; doi:10.18632/oncotarget.27715)
Supplement: Supplementary file 1 [file oncotarget-11-3405-s001.pdf]

Association of functional *IL16* polymorphisms with cancer and cardiovascular disease: a meta-analysis

SUPPLEMENTARY MATERIALS

A - All cancer and T vs G comparison for rs11556218.

Study	Experimental Events	Experimental Total	Control Events	Control Total	Weight (fixed)	Weight (random)	Odds Ratio MH, Fixed + Random, 95% CI
Shih 2020	381	1916	291	1916	14.3%	8.8%	1.39 [1.17; 1.64]
Wu 2020	183	716	199	1432	6.1%	7.8%	2.13 [1.70; 2.66]
He 2018	195	958	193	966	9.4%	7.8%	1.02 [0.82; 1.28]
Li 2018	340	1076	303	1250	11.8%	8.5%	1.44 [1.20; 1.73]
MaiMaiTiMin 2016	157	460	86	460	3.5%	6.3%	2.25 [1.66; 3.05]
Tang 2016	212	716	183	804	7.5%	7.7%	1.43 [1.13; 1.80]
Yang 2016	140	548	131	548	6.0%	6.8%	1.09 [0.83; 1.44]
Wang 2015	99	362	138	556	4.9%	6.4%	1.14 [0.84; 1.54]
Luo 2014	133	432	132	550	4.9%	6.7%	1.41 [1.06; 1.87]
Qin 2014	49	150	32	150	1.3%	3.6%	1.79 [1.06; 3.01]
Zhang 2013	250	694	232	692	9.1%	7.8%	1.12 [0.89; 1.39]
Li 2011	106	412	130	528	5.2%	6.5%	1.06 [0.79; 1.43]
Gao 2009	121	412	175	746	5.4%	6.9%	1.36 [1.03; 1.78]
Gao 2008	387	1192	233	960	10.7%	8.4%	1.50 [1.24; 1.82]
**Total (fixed effect, 95% CI)**		**10044**		**11558**	**100.0%**	**--**	**1.38 [1.30; 1.47]**
**Total (random effects, 95% CI)**					**--**	**100.0%**	**1.38 [1.23; 1.56]**

Heterogeneity: $Tau^2 = 0.0357$; $Chi^2 = 44.09$, df = 13 ($P < 0.01$); $I^2 = 71\%$

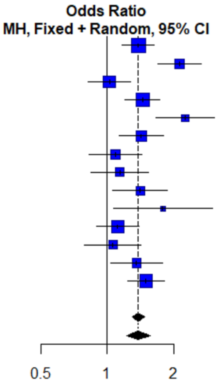

B - All cancer and TT vs TG comparison for rs11556218.

Study	Experimental Events	Experimental Total	Control Events	Control Total	Weight (fixed)	Weight (random)	Odds Ratio MH, Fixed + Random, 95% CI
Shih 2020	269	902	239	932	17.3%	9.4%	1.23 [1.00; 1.51]
Wu 2020	131	332	161	697	6.6%	8.1%	2.17 [1.64; 2.88]
He 2018	151	457	157	465	10.9%	8.2%	0.97 [0.74; 1.27]
Li 2018	252	494	247	597	11.5%	8.8%	1.48 [1.16; 1.88]
MaiMaiTiMin 2016	67	185	50	212	3.1%	5.7%	1.84 [1.19; 2.85]
Tang 2016	174	339	151	386	7.2%	7.8%	1.64 [1.22; 2.20]
Yang 2016	110	259	107	262	6.4%	6.9%	1.07 [0.75; 1.52]
Wang 2015	75	169	108	263	4.9%	6.3%	1.15 [0.77; 1.69]
Luo 2014	119	209	114	266	4.5%	6.7%	1.76 [1.22; 2.54]
Qin 2014	37	69	26	72	1.2%	3.3%	2.05 [1.04; 4.02]
Zhang 2013	114	279	112	286	6.9%	7.1%	1.07 [0.77; 1.50]
Li 2011	62	184	78	238	4.7%	6.0%	1.04 [0.69; 1.57]
Gao 2009	109	200	151	361	5.1%	7.0%	1.67 [1.18; 2.36]
Gao 2008	331	568	197	462	9.5%	8.7%	1.88 [1.47; 2.41]
**Total (fixed effect, 95% CI)**		**4646**		**5499**	**100.0%**	**--**	**1.42 [1.31; 1.55]**
**Total (random effects, 95% CI)**					**--**	**100.0%**	**1.43 [1.24; 1.66]**

Heterogeneity: $Tau^2 = 0.0468$; $Chi^2 = 37.13$, df = 13 ($P < 0.01$); $I^2 = 65\%$

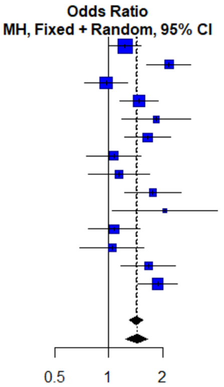

C - All cancer and TG vs GG comparison for rs11556218.

Study	Experimental Events	Experimental Total	Control Events	Control Total	Weight (fixed)	Weight (random)	Odds Ratio MH, Fixed + Random, 95% CI
Shih 2020	56	325	26	265	10.5%	12.4%	1.91 [1.16; 3.14]
Wu 2020	26	157	19	180	6.5%	7.6%	1.68 [0.89; 3.17]
He 2018	22	173	18	175	6.9%	7.0%	1.27 [0.66; 2.46]
Li 2018	44	296	28	275	10.9%	12.0%	1.54 [0.93; 2.55]
MaiMaiTiMin 2016	45	112	18	68	5.9%	7.0%	1.87 [0.97; 3.60]
Tang 2016	19	193	16	167	6.8%	6.2%	1.03 [0.51; 2.07]
Yang 2016	15	125	12	119	4.8%	4.7%	1.22 [0.54; 2.72]
Wang 2015	12	87	15	123	4.7%	4.6%	1.15 [0.51; 2.60]
Luo 2014	7	126	9	123	3.8%	2.9%	0.75 [0.27; 2.07]
Qin 2014	6	43	3	29	1.4%	1.4%	1.41 [0.32; 6.14]
Zhang 2013	68	182	60	172	17.0%	16.2%	1.11 [0.72; 1.72]
Li 2011	22	84	26	104	7.6%	7.0%	1.06 [0.55; 2.06]
Gao 2009	6	115	12	163	4.1%	3.0%	0.69 [0.25; 1.90]
Gao 2008	28	359	18	215	9.2%	8.0%	0.93 [0.50; 1.72]
**Total (fixed effect, 95% CI)**		**2377**		**2178**	**100.0%**	**--**	**1.29 [1.08; 1.53]**
**Total (random effects, 95% CI)**					**--**	**100.0%**	**1.28 [1.08; 1.53]**

Heterogeneity: $Tau^2 = 0$; $Chi^2 = 9.72$, df = 13 ($P = 0.72$); $I^2 = 0\%$

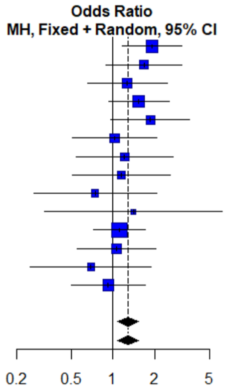

D - All cancer and TT vs GG comparison for rs11556218.

Study	Experimental Events	Experimental Total	Control Events	Control Total	Weight (fixed)	Weight (random)	Odds Ratio MH, Fixed + Random, 95% CI
Shih 2020	56	689	26	719	11.2%	10.3%	2.36 [1.46; 3.80]
Wu 2020	26	227	19	555	4.7%	8.0%	3.65 [1.98; 6.74]
He 2018	22	328	18	326	8.1%	7.6%	1.23 [0.65; 2.34]
Li 2018	44	286	28	378	9.8%	9.8%	2.27 [1.38; 3.75]
MaiMaiTiMin 2016	45	163	18	180	5.9%	8.2%	3.43 [1.89; 6.23]
Tang 2016	19	184	16	251	5.8%	6.9%	1.69 [0.84; 3.39]
Yang 2016	15	164	12	167	5.2%	5.8%	1.30 [0.59; 2.87]
Wang 2015	12	106	15	170	4.9%	5.7%	1.32 [0.59; 2.94]
Luo 2014	7	97	9	161	3.0%	4.0%	1.31 [0.47; 3.65]
Qin 2014	6	38	3	49	1.1%	2.2%	2.88 [0.67; 12.35]
Zhang 2013	68	233	60	234	20.3%	11.7%	1.20 [0.80; 1.80]
Li 2011	22	144	26	186	9.2%	8.0%	1.11 [0.60; 2.05]
Gao 2009	6	97	12	222	3.3%	4.0%	1.15 [0.42; 3.17]
Gao 2008	28	265	18	283	7.5%	7.9%	1.74 [0.94; 3.23]
**Total (fixed effect, 95% CI)**		**3021**		**3881**	**100.0%**	**--**	**1.77 [1.50; 2.10]**
**Total (random effects, 95% CI)**					**--**	**100.0%**	**1.77 [1.40; 2.22]**

Heterogeneity: $Tau^2 = 0.0736$; $Chi^2 = 22.01$, df = 13 (P = 0.06); $I^2 = 41\%$

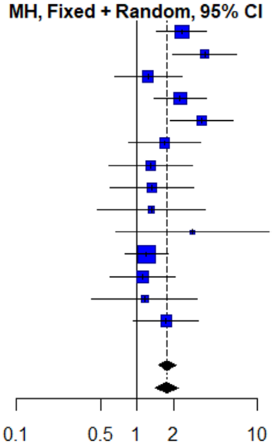

E - All cancer and TT vs TG + GG comparison for rs11556218.

Study	Experimental Events	Experimental Total	Control Events	Control Total	Weight (fixed)	Weight (random)	Odds Ratio MH, Fixed + Random, 95% CI
Shih 2020	325	958	265	958	17.0%	9.1%	1.34 [1.11; 1.63]
Wu 2020	157	358	180	716	6.5%	8.0%	2.33 [1.78; 3.04]
He 2018	173	479	175	483	10.8%	8.0%	1.00 [0.76; 1.29]
Li 2018	296	538	275	625	11.1%	8.5%	1.56 [1.23; 1.96]
MaiMaiTiMin 2016	112	230	68	230	3.4%	6.3%	2.26 [1.54; 3.32]
Tang 2016	193	358	167	402	7.0%	7.7%	1.65 [1.23; 2.19]
Yang 2016	125	274	119	274	6.3%	6.9%	1.09 [0.78; 1.53]
Wang 2015	87	181	123	278	4.9%	6.4%	1.17 [0.80; 1.70]
Luo 2014	126	216	123	275	4.4%	6.6%	1.73 [1.21; 2.48]
Qin 2014	43	75	29	75	1.2%	3.5%	2.13 [1.11; 4.09]
Zhang 2013	182	347	172	346	8.0%	7.5%	1.12 [0.83; 1.50]
Li 2011	84	206	104	264	5.2%	6.4%	1.06 [0.73; 1.54]
Gao 2009	115	206	163	373	5.0%	6.8%	1.63 [1.16; 2.29]
Gao 2008	359	596	215	480	9.2%	8.3%	1.87 [1.46; 2.38]
**Total (fixed effect, 95% CI)**		**5022**		**5779**	**100.0%**	**--**	**1.48 [1.37; 1.60]**
**Total (random effects, 95% CI)**					**--**	**100.0%**	**1.49 [1.28; 1.72]**

Heterogeneity: $Tau^2 = 0.0529$; $Chi^2 = 42.94$, df = 13 (P < 0.01); $I^2 = 70\%$

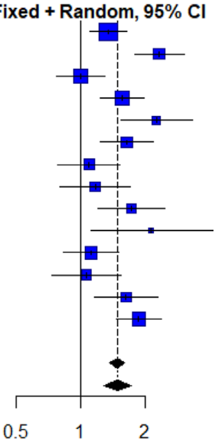

F - All cancer and TT + TG vs GG comparison for rs11556218.

Study	Experimental Events	Experimental Total	Control Events	Control Total	Weight (fixed)	Weight (random)	Odds Ratio MH, Fixed + Random, 95% CI
Shih 2020	56	958	26	958	10.4%	10.7%	2.23 [1.39; 3.58]
Wu 2020	26	358	19	716	5.0%	7.8%	2.87 [1.57; 5.27]
He 2018	22	479	18	483	7.3%	7.3%	1.24 [0.66; 2.35]
Li 2018	44	538	28	625	10.1%	10.3%	1.90 [1.17; 3.10]
MaiMaiTiMin 2016	45	230	18	230	6.2%	8.3%	2.86 [1.60; 5.12]
Tang 2016	19	358	16	402	6.1%	6.6%	1.35 [0.68; 2.67]
Yang 2016	15	274	12	274	4.8%	5.4%	1.26 [0.58; 2.75]
Wang 2015	12	181	15	278	4.7%	5.3%	1.24 [0.57; 2.72]
Luo 2014	7	216	9	275	3.3%	3.5%	0.99 [0.36; 2.70]
Qin 2014	6	75	3	75	1.2%	1.9%	2.09 [0.50; 8.67]
Zhang 2013	68	347	60	346	20.6%	13.4%	1.16 [0.79; 1.71]
Li 2011	22	206	26	264	8.7%	7.9%	1.09 [0.60; 1.99]
Gao 2009	6	206	12	373	3.5%	3.6%	0.90 [0.33; 2.44]
Gao 2008	28	596	18	480	8.1%	7.8%	1.27 [0.69; 2.32]
**Total (fixed effect, 95% CI)**		**5022**		**5779**	**100.0%**	**--**	**1.56 [1.33; 1.84]**
**Total (random effects, 95% CI)**					**--**	**100.0%**	**1.55 [1.26; 1.90]**

Heterogeneity: $Tau^2 = 0.0414$; $Chi^2 = 18.32$, df = 13 (P = 0.15); $I^2 = 29\%$

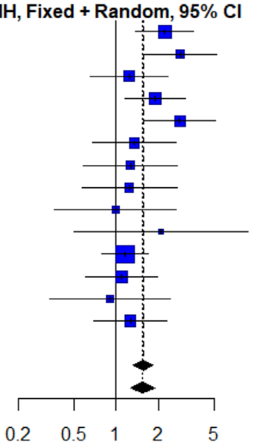

G - All cancer and TT + GG vs TG comparison for rs11556218.

Study	Experimental Events	Experimental Total	Control Events	Control Total	Weight (fixed)	Weight (random)	Odds Ratio MH, Fixed + Random, 95% CI
Shih 2020	269	958	239	958	16.9%	9.6%	1.17 [0.96; 1.44]
Wu 2020	131	358	161	716	6.7%	8.1%	1.99 [1.51; 2.63]
He 2018	151	479	157	483	10.5%	8.2%	0.96 [0.73; 1.25]
Li 2018	252	538	247	625	11.9%	9.0%	1.35 [1.07; 1.70]
MaiMaiTiMin 2016	67	230	50	230	3.5%	5.6%	1.48 [0.97; 2.26]
Tang 2016	174	358	151	402	7.2%	7.9%	1.57 [1.18; 2.10]
Yang 2016	110	274	107	274	6.3%	6.9%	1.05 [0.74; 1.47]
Wang 2015	75	181	108	278	4.9%	6.2%	1.11 [0.76; 1.63]
Luo 2014	119	216	114	275	4.4%	6.6%	1.73 [1.21; 2.48]
Qin 2014	37	75	26	75	1.3%	3.2%	1.84 [0.95; 3.54]
Zhang 2013	114	347	112	346	7.4%	7.3%	1.02 [0.74; 1.40]
Li 2011	62	206	78	264	4.7%	5.9%	1.03 [0.69; 1.53]
Gao 2009	109	206	151	373	5.0%	6.9%	1.65 [1.17; 2.33]
Gao 2008	331	596	197	480	9.5%	8.8%	1.79 [1.41; 2.29]
**Total (fixed effect, 95% CI)**		**5022**		**5779**	**100.0%**	**--**	**1.35 [1.25; 1.47]**
**Total (random effects, 95% CI)**					**--**	**100.0%**	**1.36 [1.19; 1.55]**

Heterogeneity: $Tau^2 = 0.0389$; $Chi^2 = 33.91$, df = 13 ($P < 0.01$); $I^2 = 62\%$

Odds Ratio MH, Fixed + Random, 95% CI

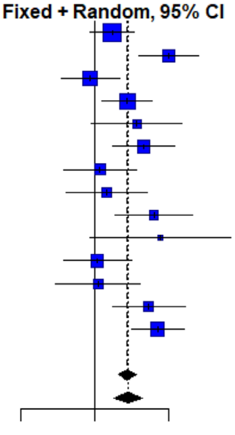

H - Gastric cancer and T vs C comparison for rs4778889.

Study	Experimental Events	Experimental Total	Control Events	Control Total	Weight (fixed)	Weight (random)	Odds Ratio MH, Fixed + Random, 95% CI
He 2018	242	958	242	966	45.9%	37.9%	1.01 [0.82; 1.24]
Zhang 2013	204	694	164	694	29.5%	32.6%	1.35 [1.06; 1.71]
Gao 2008	116	440	208	960	24.5%	29.5%	1.29 [1.00; 1.68]
**Total (fixed effect, 95% CI)**		**2092**		**2620**	**100.0%**	**--**	**1.18 [1.03; 1.35]**
**Total (random effects, 95% CI)**					**--**	**100.0%**	**1.19 [0.99; 1.44]**

Heterogeneity: $Tau^2 = 0.0128$; $Chi^2 = 3.79$, df = 2 ($P = 0.15$); $I^2 = 47\%$

Odds Ratio MH, Fixed + Random, 95% CI

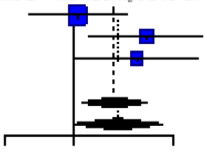

I - Gastric cancer and TT vs CC comparison for rs4778889.

Study	Experimental Events	Experimental Total	Control Events	Control Total	Weight (fixed)	Weight (random)	Odds Ratio MH, Fixed + Random, 95% CI
He 2018	30	297	25	291	39.7%	35.5%	1.20 [0.68; 2.09]
Zhang 2013	45	233	29	241	40.2%	43.1%	1.75 [1.05; 2.90]
Gao 2008	13	130	22	316	20.2%	21.4%	1.48 [0.72; 3.05]
**Total (fixed effect, 95% CI)**		**660**		**848**	**100.0%**	**--**	**1.48 [1.06; 2.06]**
**Total (random effects, 95% CI)**					**--**	**100.0%**	**1.48 [1.06; 2.06]**

Heterogeneity: $Tau^2 = 0$; $Chi^2 = 0.98$, df = 2 ($P = 0.61$); $I^2 = 0\%$

Odds Ratio MH, Fixed + Random, 95% CI

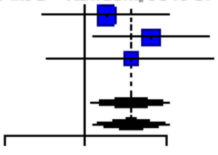

J - Gastric cancer and TT + TC vs CC comparison for rs4778889.

Study	Experimental Events	Experimental Total	Control Events	Control Total	Weight (fixed)	Weight (random)	Odds Ratio MH, Fixed + Random, 95% CI
He 2018	30	479	25	483	37.9%	35.3%	1.22 [0.71; 2.11]
Zhang 2013	45	347	29	347	41.0%	43.5%	1.63 [1.00; 2.67]
Gao 2008	13	220	22	480	21.1%	21.2%	1.31 [0.65; 2.65]
**Total (fixed effect, 95% CI)**		**1046**		**1310**	**100.0%**	**--**	**1.41 [1.02; 1.95]**
**Total (random effects, 95% CI)**					**--**	**100.0%**	**1.41 [1.02; 1.95]**

Heterogeneity: $Tau^2 = 0$; $Chi^2 = 0.65$, df = 2 ($P = 0.72$); $I^2 = 0\%$

Odds Ratio MH, Fixed + Random, 95% CI

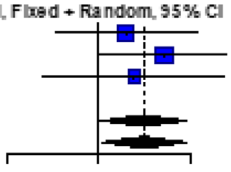

K - Cardiovascular disease and T vs G comparison for rs11556218

Study	Experimental Events	Experimental Total	Control Events	Control Total	Weight (fixed)	Weight (random)	Odds Ratio MH, Fixed + Random, 95% CI
Yang 2018	139	476	118	356	18.8%	16.4%	0.83 [0.62; 1.12]
Liu 2013	118	396	106	472	13.3%	16.3%	1.47 [1.08; 1.99]
Tong 2013	137	652	104	680	15.8%	16.6%	1.47 [1.11; 1.95]
Chen 2011 (1)	281	600	181	794	16.3%	17.3%	2.98 [2.37; 3.76]
Chen 2011 (2)	288	848	164	664	23.8%	17.3%	1.57 [1.25; 1.97]
Wu 2011	100	314	103	404	12.0%	16.0%	1.37 [0.99; 1.89]
**Total (fixed effect, 95% CI)**		**3286**		**3370**	**100.0%**	**--**	**1.61 [1.44; 1.79]**
**Total (random effects, 95% CI)**					**--**	**100.0%**	**1.51 [1.07; 2.14]**

Heterogeneity: $Tau^2 = 0.1669$; $Chi^2 = 48.33$, df = 5 ($P < 0.01$); $I^2 = 90\%$

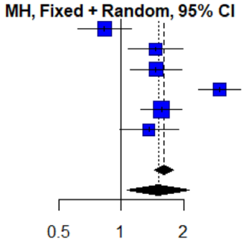

L - Cardiovascular disease and TT vs TG comparison for rs11556218

Study	Experimental Events	Experimental Total	Control Events	Control Total	Weight (fixed)	Weight (random)	Odds Ratio MH, Fixed + Random, 95% CI
Yang 2018	71	204	46	142	14.7%	16.3%	1.11 [0.71; 1.76]
Liu 2013	102	190	94	230	16.4%	16.7%	1.68 [1.14; 2.47]
Tong 2013	69	292	80	328	24.0%	16.8%	0.96 [0.66; 1.39]
Chen 2011 (1)	235	277	149	381	7.9%	16.7%	8.71 [5.91; 12.84]
Chen 2011 (2)	264	412	144	322	24.2%	17.1%	2.20 [1.64; 2.97]
Wu 2011	92	153	87	194	12.7%	16.4%	1.85 [1.21; 2.85]
**Total (fixed effect, 95% CI)**		**1528**		**1597**	**100.0%**	**--**	**2.13 [1.84; 2.47]**
**Total (random effects, 95% CI)**					**--**	**100.0%**	**2.00 [1.08; 3.71]**

Heterogeneity: $Tau^2 = 0.5535$; $Chi^2 = 78.41$, df = 5 ($P < 0.01$); $I^2 = 94\%$

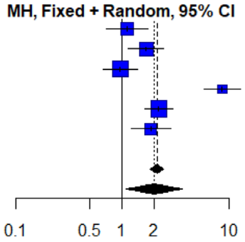

M - Cardiovascular disease and TT vs TG+GG comparison for rs11556218

Study	Experimental Events	Experimental Total	Control Events	Control Total	Weight (fixed)	Weight (random)	Odds Ratio MH, Fixed + Random, 95% CI
Yang 2018	105	238	82	178	19.6%	16.5%	0.92 [0.63; 1.37]
Liu 2013	110	198	100	236	15.2%	16.6%	1.70 [1.16; 2.49]
Tong 2013	103	326	92	340	23.1%	16.9%	1.25 [0.89; 1.74]
Chen 2011 (1)	258	300	165	397	7.4%	16.6%	8.64 [5.89; 12.66]
Chen 2011 (2)	276	424	154	332	22.6%	17.1%	2.16 [1.61; 2.89]
Wu 2011	96	157	95	202	12.1%	16.3%	1.77 [1.16; 2.71]
**Total (fixed effect, 95% CI)**		**1643**		**1685**	**100.0%**	**--**	**2.07 [1.80; 2.39]**
**Total (random effects, 95% CI)**					**--**	**100.0%**	**2.00 [1.11; 3.63]**

Heterogeneity: $Tau^2 = 0.5154$; $Chi^2 = 80.52$, df = 5 ($P < 0.01$); $I^2 = 94\%$

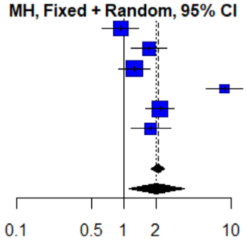

N - Cardiovascular disease and (TT+GG vs TG) comparison for rs11556218

Study	Experimental Events	Experimental Total	Control Events	Control Total	Weight (fixed)	Weight (random)	Odds Ratio MH, Fixed + Random, 95% CI
Yang 2018	71	238	46	178	14.2%	16.2%	1.22 [0.79; 1.89]
Liu 2013	102	198	94	236	16.0%	16.6%	1.61 [1.10; 2.35]
Tong 2013	69	326	80	340	23.7%	16.7%	0.87 [0.61; 1.26]
Chen 2011 (1)	235	300	149	397	10.7%	16.9%	6.02 [4.28; 8.47]
Chen 2011 (2)	264	424	144	332	23.4%	17.2%	2.15 [1.61; 2.89]
Wu 2011	92	157	87	202	12.1%	16.3%	1.87 [1.23; 2.85]
**Total (fixed effect, 95% CI)**		**1643**		**1685**	**100.0%**	**--**	**2.01 [1.74; 2.32]**
**Total (random effects, 95% CI)**					**--**	**100.0%**	**1.87 [1.08; 3.23]**

Heterogeneity: $Tau^2 = 0.4281$; $Chi^2 = 66.33$, df = 5 ($P < 0.01$); $I^2 = 92\%$

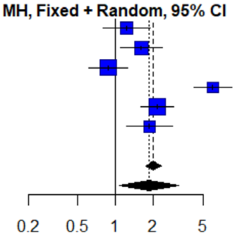

Supplementary Figure 1: Forest plot of comparisons for all polymorphisms associated with disease in the pooled studies.

A - Cancer and T vs G comparison for rs11556218

Study or Subgroup	Experimental Events	Experimental Total	Control Events	Control Total	Weight (fixed)	Weight (random)	Odds Ratio MH, Fixed + Random, 95% CI
Type = Carcinoma and others							
Shih 2020	381	1916	291	1916	15.1%	9.0%	1.39 [1.17; 1.64]
Wu 2020	183	716	199	1432	6.4%	7.9%	2.13 [1.70; 2.66]
Li 2018	340	1076	303	1250	12.4%	8.7%	1.44 [1.20; 1.73]
MaiMaiTiMin 2016	157	460	86	460	3.7%	6.4%	2.25 [1.66; 3.05]
Tang 2016	212	716	183	804	7.9%	7.8%	1.43 [1.13; 1.80]
Luo 2014	133	432	132	550	5.2%	6.7%	1.41 [1.06; 1.87]
Qin 2014	49	150	32	150	1.4%	3.5%	1.79 [1.06; 3.01]
Li 2011	106	412	130	528	5.5%	6.5%	1.06 [0.79; 1.43]
Gao 2009	121	412	175	746	5.7%	6.9%	1.36 [1.03; 1.78]
Gao 2008 (CRC)	247	752	233	960	8.9%	8.1%	1.53 [1.23; 1.89]
Total (fixed effect, 95% CI)		7042		8796	72.2%	--	1.51 [1.40; 1.63]
Total (random effects, 95% CI)					--	71.4%	1.53 [1.34; 1.73]
Heterogeneity: $Tau^2 = 0.0246$; $Chi^2 = 23.76$, df = 9 (P < 0.01); $I^2 = 62\%$							
Type = Gastric and renal cancer							
He 2018	195	958	193	966	9.9%	7.9%	1.02 [0.82; 1.28]
Yang 2016	140	548	131	548	6.3%	6.9%	1.09 [0.83; 1.44]
Wang 2015	99	362	138	556	5.1%	6.4%	1.14 [0.84; 1.54]
Gao 2008 (GC)	140	440	233	960	6.5%	7.4%	1.46 [1.14; 1.87]
Total (fixed effect, 95% CI)		2308		3030	27.8%	--	1.16 [1.02; 1.32]
Total (random effects, 95% CI)					--	28.6%	1.17 [0.99; 1.37]
Heterogeneity: $Tau^2 = 0.0094$; $Chi^2 = 4.61$, df = 3 (P = 0.20); $I^2 = 35\%$							
**Total (fixed effect, 95% CI)**		**9350**		**11826**	**100.0%**	**--**	**1.41 [1.32; 1.51]**
**Total (random effects, 95% CI)**					**--**	**100.0%**	**1.41 [1.26; 1.59]**
Heterogeneity: $Tau^2 = 0.0326$; $Chi^2 = 40.32$, df = 13 (P < 0.01); $I^2 = 68\%$							

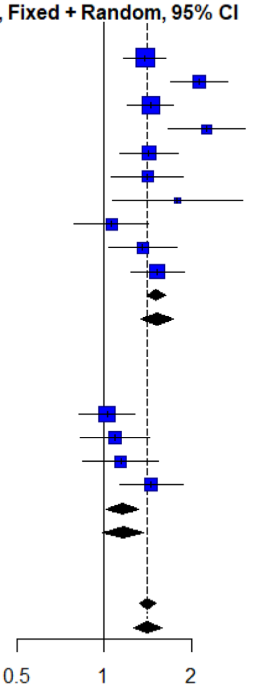

B - Cancer and TT vs TG comparison for rs11556218

Study or Subgroup	Experimental Events	Experimental Total	Control Events	Control Total	Weight (fixed)	Weight (random)	Odds Ratio MH, Fixed + Random, 95% CI
Type = Carcinoma and others							
Shih 2020	269	902	239	932	17.9%	9.5%	1.23 [1.00; 1.51]
Wu 2020	131	332	161	697	6.8%	8.1%	2.17 [1.64; 2.88]
Li 2018	252	494	247	597	11.9%	8.9%	1.48 [1.16; 1.88]
MaiMaiTiMin 2016	67	185	50	212	3.2%	5.7%	1.84 [1.19; 2.85]
Tang 2016	174	339	151	386	7.5%	7.9%	1.64 [1.22; 2.20]
Luo 2014	119	209	114	266	4.7%	6.7%	1.76 [1.22; 2.54]
Qin 2014	37	69	26	72	1.3%	3.3%	2.05 [1.04; 4.02]
Li 2011	62	184	78	238	4.9%	6.1%	1.04 [0.69; 1.57]
Gao 2009	109	200	151	361	5.3%	7.0%	1.67 [1.18; 2.36]
Gao 2008 (CRC)	219	362	197	462	7.4%	8.1%	2.06 [1.56; 2.73]
Total (fixed effect, 95% CI)		3276		4223	70.9%	--	1.59 [1.44; 1.75]
Total (random effects, 95% CI)					--	71.2%	1.63 [1.40; 1.89]
Heterogeneity: $Tau^2 = 0.0301$; $Chi^2 = 19.77$, df = 9 (P = 0.02); $I^2 = 54\%$							
Type = Gastric and renal cancer							
He 2018	151	457	157	465	11.3%	8.3%	0.97 [0.74; 1.27]
Yang 2016	110	259	107	262	6.6%	7.0%	1.07 [0.75; 1.52]
Wang 2015	75	169	108	263	5.1%	6.3%	1.15 [0.77; 1.69]
Gao 2008 (GC)	112	206	197	462	6.0%	7.3%	1.60 [1.15; 2.23]
Total (fixed effect, 95% CI)		1091		1452	29.1%	--	1.15 [0.98; 1.36]
Total (random effects, 95% CI)					--	28.8%	1.17 [0.93; 1.46]
Heterogeneity: $Tau^2 = 0.0244$; $Chi^2 = 5.56$, df = 3 (P = 0.13); $I^2 = 46\%$							
**Total (fixed effect, 95% CI)**		**4367**		**5675**	**100.0%**	**--**	**1.46 [1.35; 1.59]**
**Total (random effects, 95% CI)**					**--**	**100.0%**	**1.48 [1.28; 1.71]**
Heterogeneity: $Tau^2 = 0.0463$; $Chi^2 = 36.38$, df = 13 (P < 0.01); $I^2 = 64\%$							

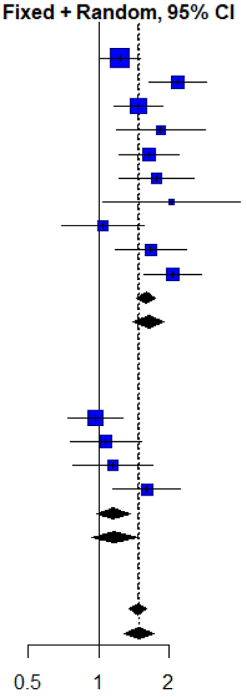

C - Cancer and TG vs GG comparison for rs11556218

Study or Subgroup	Experimental Events	Experimental Total	Control Events	Control Total	Weight (fixed)	Weight (random)	Odds Ratio MH, Fixed + Random, 95% CI
Type = Carcinoma and others							
Shih 2020	56	325	26	265	12.0%	14.2%	1.91 [1.16; 3.14]
Wu 2020	26	157	19	180	7.5%	8.7%	1.68 [0.89; 3.17]
Li 2018	44	296	28	275	12.6%	13.7%	1.54 [0.93; 2.55]
MaiMaiTiMin 2016	45	112	18	68	6.8%	8.1%	1.87 [0.97; 3.60]
Tang 2016	19	193	16	167	7.9%	7.1%	1.03 [0.51; 2.07]
Luo 2014	7	126	9	123	4.4%	3.4%	0.75 [0.27; 2.07]
Qin 2014	6	43	3	29	1.6%	1.6%	1.41 [0.32; 6.14]
Li 2011	22	84	26	104	8.7%	8.1%	1.06 [0.55; 2.06]
Gao 2009	6	115	12	163	4.8%	3.4%	0.69 [0.25; 1.90]
Gao 2008 (CRC)	14	233	18	215	8.9%	6.7%	0.70 [0.34; 1.44]
Total (fixed effect, 95% CI)		1684		1589	75.1%	--	1.33 [1.08; 1.65]
Total (random effects, 95% CI)					--	74.9%	1.30 [1.02; 1.66]
Heterogeneity: Tau^2 = 0.0243; Chi^2 = 10.74, df = 9 (P = 0.29); I^2 = 16%							
Type = Gastric and renal cancer							
He 2018	22	173	18	175	7.9%	8.0%	1.27 [0.66; 2.46]
Yang 2016	15	125	12	119	5.5%	5.4%	1.22 [0.54; 2.72]
Wang 2015	12	87	15	123	5.4%	5.3%	1.15 [0.51; 2.60]
Gao 2008 (GC)	14	126	18	215	6.0%	6.5%	1.37 [0.66; 2.86]
Total (fixed effect, 95% CI)		511		632	24.9%	--	1.26 [0.86; 1.82]
Total (random effects, 95% CI)					--	25.1%	1.26 [0.87; 1.83]
Heterogeneity: Tau^2 = 0; Chi^2 = 0.1, df = 3 (P = 0.99); I^2 = 0%							
**Total (fixed effect, 95% CI)**		**2195**		**2221**	**100.0%**	**--**	**1.31 [1.09; 1.58]**
**Total (random effects, 95% CI)**					**--**	**100.0%**	**1.31 [1.09; 1.58]**
Heterogeneity: Tau^2 = 0; Chi^2 = 10.91, df = 13 (P = 0.62); I^2 = 0%							

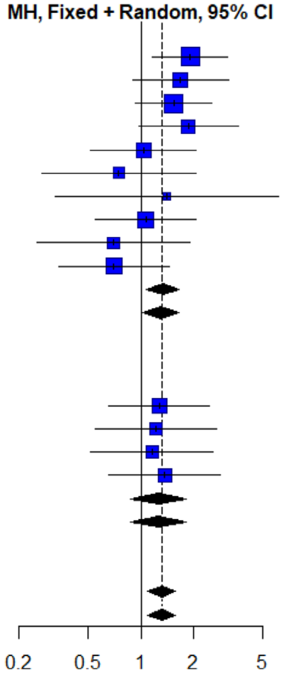

D - Cancer and TT vs GG comparison for rs11556218

Study or Subgroup	Experimental Events	Experimental Total	Control Events	Control Total	Weight (fixed)	Weight (random)	Odds Ratio MH, Fixed + Random, 95% CI
Type = Carcinoma and others							
Shih 2020	56	689	26	719	13.7%	11.7%	2.36 [1.46; 3.80]
Wu 2020	26	227	19	555	5.7%	8.6%	3.65 [1.98; 6.74]
Li 2018	44	286	28	378	11.9%	11.1%	2.27 [1.38; 3.75]
MaiMaiTiMin 2016	45	163	18	180	7.2%	9.0%	3.43 [1.89; 6.23]
Tang 2016	19	184	16	251	7.1%	7.3%	1.69 [0.84; 3.39]
Luo 2014	7	97	9	161	3.7%	4.0%	1.31 [0.47; 3.65]
Qin 2014	6	38	3	49	1.3%	2.1%	2.88 [0.67; 12.35]
Li 2011	22	144	26	186	11.3%	8.6%	1.11 [0.60; 2.05]
Gao 2009	6	97	12	222	4.0%	4.1%	1.15 [0.42; 3.17]
Gao 2008 (CRC)	14	157	18	283	6.8%	6.8%	1.44 [0.70; 2.98]
Total (fixed effect, 95% CI)		2082		2984	72.8%	--	2.10 [1.71; 2.58]
Total (random effects, 95% CI)					--	73.3%	2.04 [1.56; 2.67]
Heterogeneity: Tau^2 = 0.0644; Chi^2 = 13.93, df = 9 (P = 0.12); I^2 = 35%							
Type = Gastric and renal cancer							
He 2018	22	328	18	326	9.9%	8.1%	1.23 [0.65; 2.34]
Yang 2016	15	164	12	167	6.3%	6.0%	1.30 [0.59; 2.87]
Wang 2015	12	106	15	170	6.0%	5.9%	1.32 [0.59; 2.94]
Gao 2008 (GC)	14	108	18	283	5.1%	6.7%	2.19 [1.05; 4.58]
Total (fixed effect, 95% CI)		706		946	27.2%	--	1.45 [1.00; 2.09]
Total (random effects, 95% CI)					--	26.7%	1.46 [1.01; 2.11]
Heterogeneity: Tau^2 = 0; Chi^2 = 1.59, df = 3 (P = 0.66); I^2 = 0%							
**Total (fixed effect, 95% CI)**		**2788**		**3930**	**100.0%**	**--**	**1.92 [1.60; 2.30]**
**Total (random effects, 95% CI)**					**--**	**100.0%**	**1.87 [1.50; 2.34]**
Heterogeneity: Tau^2 = 0.0503; Chi^2 = 18.33, df = 13 (P = 0.15); I^2 = 29%							

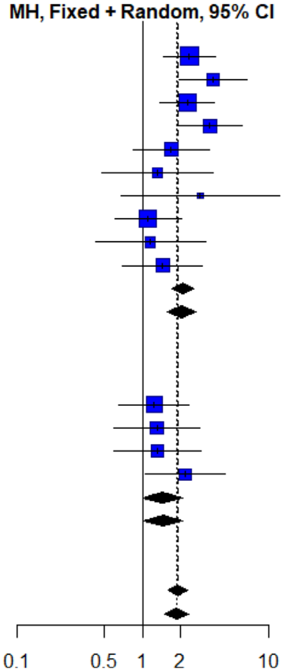

E - Cancer and TT vs TG+GG comparison for rs11556218

Study or Subgroup	Experimental Events	Experimental Total	Control Events	Control Total	Weight (fixed)	Weight (random)	Odds Ratio MH, Fixed + Random, 95% CI
Type = Carcinoma and others							
Shih 2020	325	958	265	958	17.8%	9.2%	1.34 [1.11; 1.63]
Wu 2020	157	358	180	716	6.9%	8.0%	2.33 [1.78; 3.04]
Li 2018	296	538	275	625	11.6%	8.6%	1.56 [1.23; 1.96]
MaiMaiTiMin 2016	112	230	68	230	3.5%	6.3%	2.26 [1.54; 3.32]
Tang 2016	193	358	167	402	7.4%	7.7%	1.65 [1.23; 2.19]
Luo 2014	126	216	123	275	4.6%	6.6%	1.73 [1.21; 2.48]
Qin 2014	43	75	29	75	1.3%	3.5%	2.13 [1.11; 4.09]
Li 2011	84	206	104	264	5.5%	6.5%	1.06 [0.73; 1.54]
Gao 2009	115	206	163	373	5.2%	6.9%	1.63 [1.16; 2.29]
Gao 2008 (CRC)	233	376	215	480	7.3%	7.9%	2.01 [1.53; 2.64]
Total (fixed effect, 95% CI)		3521		4398	71.1%	--	1.66 [1.51; 1.82]
Total (random effects, 95% CI)					--	71.3%	1.70 [1.46; 1.97]
Heterogeneity: $Tau^2 = 0.0321$; $Chi^2 = 21.49$, df = 9 (P = 0.01); $I^2 = 58\%$							
Type = Gastric and renal cancer							
He 2018	173	479	175	483	11.3%	8.1%	1.00 [0.76; 1.29]
Yang 2016	125	274	119	274	6.6%	7.0%	1.09 [0.78; 1.53]
Wang 2015	87	181	123	278	5.1%	6.4%	1.17 [0.80; 1.70]
Gao 2008 (GC)	126	220	215	480	5.9%	7.2%	1.65 [1.20; 2.28]
Total (fixed effect, 95% CI)		1154		1515	28.9%	--	1.18 [1.01; 1.38]
Total (random effects, 95% CI)					--	28.7%	1.19 [0.95; 1.50]
Heterogeneity: $Tau^2 = 0.0268$; $Chi^2 = 6.01$, df = 3 (P = 0.11); $I^2 = 50\%$							
**Total (fixed effect, 95% CI)**		**4675**		**5913**	**100.0%**	**--**	**1.52 [1.40; 1.64]**
**Total (random effects, 95% CI)**					**--**	**100.0%**	**1.54 [1.33; 1.78]**
Heterogeneity: $Tau^2 = 0.0506$; $Chi^2 = 40.73$, df = 13 (P < 0.01); $I^2 = 68\%$							

Odds Ratio MH, Fixed + Random, 95% CI

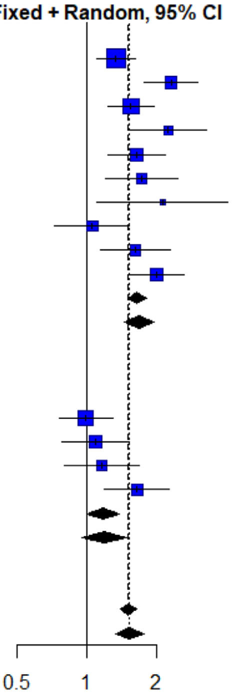

F - Cancer and TT+TG vs GG comparison for rs11556218

Study or Subgroup	Experimental Events	Experimental Total	Control Events	Control Total	Weight (fixed)	Weight (random)	Odds Ratio MH, Fixed + Random, 95% CI
Type = Carcinoma and others							
Shih 2020	56	958	26	958	12.7%	12.0%	2.23 [1.39; 3.58]
Wu 2020	26	358	19	716	6.1%	8.6%	2.87 [1.57; 5.27]
Li 2018	44	538	28	625	12.3%	11.6%	1.90 [1.17; 3.10]
MaiMaiTiMin 2016	45	230	18	230	7.5%	9.2%	2.86 [1.60; 5.12]
Tang 2016	19	358	16	402	7.4%	7.2%	1.35 [0.68; 2.67]
Luo 2014	7	216	9	275	4.0%	3.8%	0.99 [0.36; 2.70]
Qin 2014	6	75	3	75	1.4%	2.0%	2.09 [0.50; 8.67]
Li 2011	22	206	26	264	10.5%	8.7%	1.09 [0.60; 1.99]
Gao 2009	6	206	12	373	4.3%	3.8%	0.90 [0.33; 2.44]
Gao 2008 (CRC)	14	376	18	480	7.9%	6.7%	0.99 [0.49; 2.02]
Total (fixed effect, 95% CI)		3521		4398	74.1%	--	1.76 [1.44; 2.16]
Total (random effects, 95% CI)					--	73.7%	1.69 [1.28; 2.22]
Heterogeneity: $Tau^2 = 0.0726$; $Chi^2 = 14.77$, df = 9 (P = 0.10); $I^2 = 39\%$							
Type = Gastric and renal cancer							
He 2018	22	479	18	483	8.9%	8.0%	1.24 [0.66; 2.35]
Yang 2016	15	274	12	274	5.9%	5.8%	1.26 [0.58; 2.75]
Wang 2015	12	181	15	278	5.7%	5.8%	1.24 [0.57; 2.72]
Gao 2008 (GC)	14	220	18	480	5.5%	6.7%	1.74 [0.85; 3.57]
Total (fixed effect, 95% CI)		1154		1515	25.9%	--	1.35 [0.94; 1.94]
Total (random effects, 95% CI)					--	26.3%	1.36 [0.95; 1.95]
Heterogeneity: $Tau^2 = 0$; $Chi^2 = 0.62$, df = 3 (P = 0.89); $I^2 = 0\%$							
**Total (fixed effect, 95% CI)**		**4675**		**5913**	**100.0%**	**--**	**1.66 [1.39; 1.98]**
**Total (random effects, 95% CI)**					**--**	**100.0%**	**1.61 [1.31; 1.99]**
Heterogeneity: $Tau^2 = 0.0351$; $Chi^2 = 16.86$, df = 13 (P = 0.21); $I^2 = 23\%$							

Odds Ratio MH, Fixed + Random, 95% CI

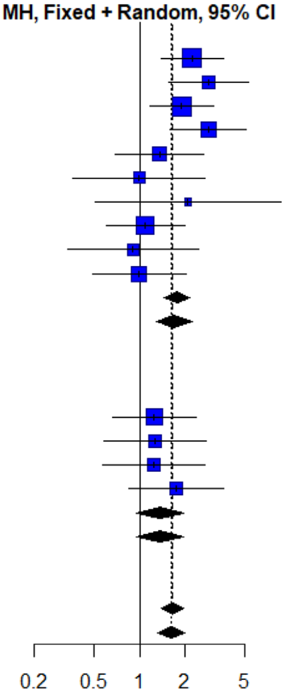

G - Cancer and TT+GG vs TG comparison for rs11556218

Study or Subgroup	Experimental Events	Experimental Total	Control Events	Control Total	Weight (fixed)	Weight (random)	Odds Ratio MH, Fixed + Random, 95% CI
Type = Carcinoma and others							
Shih 2020	269	958	239	958	17.5%	9.7%	1.17 [0.96; 1.44]
Wu 2020	131	358	161	716	6.9%	8.1%	1.99 [1.51; 2.63]
Li 2018	252	538	247	625	12.4%	9.0%	1.35 [1.07; 1.70]
MaiMaiTiMin 2016	67	230	50	230	3.6%	5.6%	1.48 [0.97; 2.26]
Tang 2016	174	358	151	402	7.5%	7.9%	1.57 [1.18; 2.10]
Luo 2014	119	216	114	275	4.6%	6.6%	1.73 [1.21; 2.48]
Qin 2014	37	75	26	75	1.3%	3.2%	1.84 [0.95; 3.54]
Li 2011	62	206	78	264	4.9%	6.0%	1.03 [0.69; 1.53]
Gao 2009	109	206	151	373	5.2%	6.9%	1.65 [1.17; 2.33]
Gao 2008 (CRC)	219	376	197	480	7.4%	8.2%	2.00 [1.52; 2.64]
Total (fixed effect, 95% CI)		3521		4398	71.3%	--	1.50 [1.36; 1.65]
Total (random effects, 95% CI)					--	71.3%	1.53 [1.32; 1.77]
Heterogeneity: $Tau^2 = 0.0283$; $Chi^2 = 19.51$, df = 9 (P = 0.02); $I^2 = 54\%$							
Type = Gastric and renal cancer							
He 2018	151	479	157	483	10.9%	8.3%	0.96 [0.73; 1.25]
Yang 2016	110	274	107	274	6.5%	6.9%	1.05 [0.74; 1.47]
Wang 2015	75	181	108	278	5.1%	6.3%	1.11 [0.76; 1.63]
Gao 2008 (GC)	112	220	197	480	6.2%	7.3%	1.49 [1.08; 2.05]
Total (fixed effect, 95% CI)		1154		1515	28.7%	--	1.12 [0.95; 1.31]
Total (random effects, 95% CI)					--	28.7%	1.13 [0.92; 1.37]
Heterogeneity: $Tau^2 = 0.0138$; $Chi^2 = 4.5$, df = 3 (P = 0.21); $I^2 = 33\%$							
**Total (fixed effect, 95% CI)**		**4675**		**5913**	**100.0%**	**--**	**1.39 [1.28; 1.51]**
**Total (random effects, 95% CI)**					**--**	**100.0%**	**1.41 [1.23; 1.61]**
Heterogeneity: $Tau^2 = 0.0391$; $Chi^2 = 33.51$, df = 13 (P < 0.01); $I^2 = 61\%$							

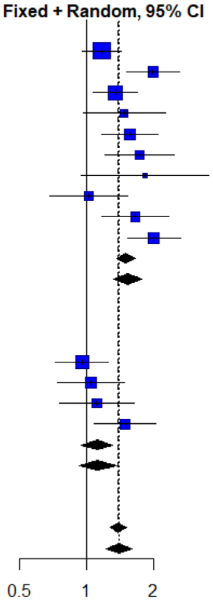

H - Cardiovascular disease and T vs G comparison for rs11556218.

Study	Experimental Events	Experimental Total	Control Events	Control Total	Weight (fixed)	Weight (random)	Odds Ratio MH, Fixed + Random, 95% CI
Liu 2013	118	396	106	472	16.4%	19.3%	1.47 [1.08; 1.99]
Tong 2013	137	652	104	680	19.4%	19.9%	1.47 [1.11; 1.95]
Chen 2001 (1)	281	600	181	794	20.0%	21.0%	2.98 [2.37; 3.76]
Chen 2001 (2)	288	848	164	664	29.3%	21.1%	1.57 [1.25; 1.97]
Wu 2011	100	314	103	404	14.8%	18.8%	1.37 [0.99; 1.89]
**Total (fixed effect, 95% CI)**		**2810**		**3014**	**100.0%**	**--**	**1.79 [1.59; 2.01]**
**Total (random effects, 95% CI)**					**--**	**100.0%**	**1.70 [1.25; 2.32]**
Heterogeneity: $Tau^2 = 0.1043$; $Chi^2 = 26.29$, df = 4 (P < 0.01); $I^2 = 85\%$							

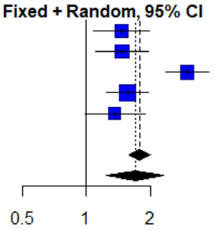

I - Cardiovascular disease and TT vs TG comparison for rs11556218.

Study	Experimental Events	Experimental Total	Control Events	Control Total	Weight (fixed)	Weight (random)	Odds Ratio MH, Fixed + Random, 95% CI
Liu 2013	102	190	94	230	19.3%	19.9%	1.68 [1.14; 2.47]
Tong 2013	69	292	80	328	28.1%	20.0%	0.96 [0.66; 1.39]
Chen 2001 (1)	235	277	149	381	9.3%	19.9%	8.71 [5.91; 12.84]
Chen 2001 (2)	264	412	144	322	28.4%	20.4%	2.20 [1.64; 2.97]
Wu 2011	92	153	87	194	14.9%	19.6%	1.85 [1.21; 2.85]
**Total (fixed effect, 95% CI)**		**1324**		**1455**	**100.0%**	**--**	**2.31 [1.97; 2.70]**
**Total (random effects, 95% CI)**					**--**	**100.0%**	**2.25 [1.12; 4.50]**
Heterogeneity: $Tau^2 = 0.5907$; $Chi^2 = 70.53$, df = 4 (P < 0.01); $I^2 = 94\%$							

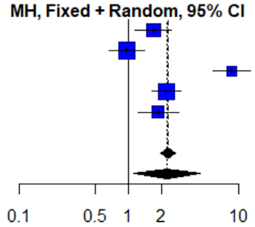

J - Cardiovascular disease and TT vs GG comparison for rs11556218.

Study	Experimental Events	Experimental Total	Control Events	Control Total	Weight (fixed)	Weight (random)	Odds Ratio MH, Fixed + Random, 95% CI
Liu 2013	8	96	6	142	13.4%	17.7%	2.06 [0.69; 6.14]
Tong 2013	34	257	12	260	31.4%	23.1%	3.15 [1.59; 6.24]
Chen 2001 (1)	23	65	16	248	13.0%	22.6%	7.94 [3.87; 16.28]
Chen 2001 (2)	12	160	10	188	25.8%	20.6%	1.44 [0.61; 3.43]
Wu 2011	4	65	8	115	16.4%	15.9%	0.88 [0.25; 3.03]
**Total (fixed effect, 95% CI)**		**643**		**953**	**100.0%**	**--**	**2.81 [1.94; 4.08]**
**Total (random effects, 95% CI)**					**--**	**100.0%**	**2.50 [1.19; 5.25]**
Heterogeneity: $Tau^2 = 0.4991$; $Chi^2 = 14.11$, df = 4 (P < 0.01); $I^2 = 72\%$							

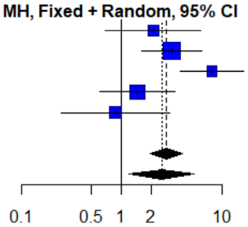

K - Cardiovascular disease and TT vs TG + GG comparison for rs11556218.

Study	Experimental Events	Experimental Total	Control Events	Control Total	Weight (fixed)	Weight (random)	Odds Ratio MH, Fixed + Random, 95% CI
Liu 2013	110	198	100	236	18.9%	19.9%	1.70 [1.16; 2.49]
Tong 2013	103	326	92	340	28.7%	20.2%	1.25 [0.89; 1.74]
Chen 2001 (1)	258	300	165	397	9.3%	19.9%	8.64 [5.89; 12.66]
Chen 2001 (2)	276	424	154	332	28.1%	20.5%	2.16 [1.61; 2.89]
Wu 2011	96	157	95	202	15.0%	19.6%	1.77 [1.16; 2.71]
**Total (fixed effect, 95% CI)**		**1405**		**1507**	**100.0%**	**--**	**2.35 [2.02; 2.74]**
**Total (random effects, 95% CI)**					**--**	**100.0%**	**2.33 [1.24; 4.41]**

Heterogeneity: $Tau^2 = 0.4923$; $Chi^2 = 63.20$, df = 4 ($P < 0.01$); $I^2 = 94\%$

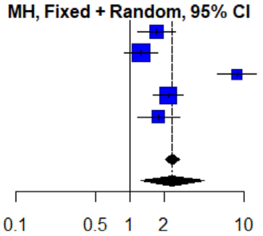

L - Cardiovascular disease and TT + TG vs GG comparison for rs11556218.

Study	Experimental Events	Experimental Total	Control Events	Control Total	Weight (fixed)	Weight (random)	Odds Ratio MH, Fixed + Random, 95% CI
Liu 2013	8	198	6	236	11.4%	15.6%	1.61 [0.55; 4.73]
Tong 2013	34	326	12	340	22.8%	25.1%	3.18 [1.62; 6.26]
Chen 2001 (1)	23	300	16	397	27.5%	25.7%	1.98 [1.03; 3.81]
Chen 2001 (2)	12	424	10	332	23.6%	20.4%	0.94 [0.40; 2.20]
Wu 2011	4	157	8	202	14.8%	13.2%	0.63 [0.19; 2.14]
**Total (fixed effect, 95% CI)**		**1405**		**1507**	**100.0%**	**--**	**1.77 [1.24; 2.53]**
**Total (random effects, 95% CI)**					**--**	**100.0%**	**1.60 [0.93; 2.73]**

Heterogeneity: $Tau^2 = 0.1796$; $Chi^2 = 7.89$, df = 4 ($P = 0.10$); $I^2 = 49\%$

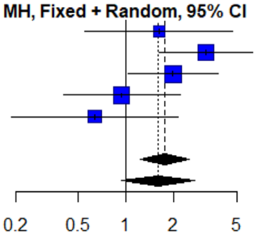

M - Cardiovascular disease and TT + GG vs TG comparison for rs11556218.

Study	Experimental Events	Experimental Total	Control Events	Control Total	Weight (fixed)	Weight (random)	Odds Ratio MH, Fixed + Random, 95% CI
Liu 2013	102	198	94	236	18.6%	19.9%	1.61 [1.10; 2.35]
Tong 2013	69	326	80	340	27.6%	20.0%	0.87 [0.61; 1.26]
Chen 2001 (1)	235	300	149	397	12.4%	20.2%	6.02 [4.28; 8.47]
Chen 2001 (2)	264	424	144	332	27.3%	20.5%	2.15 [1.61; 2.89]
Wu 2011	92	157	87	202	14.1%	19.5%	1.87 [1.23; 2.85]
**Total (fixed effect, 95% CI)**		**1405**		**1507**	**100.0%**	**--**	**2.14 [1.83; 2.49]**
**Total (random effects, 95% CI)**					**--**	**100.0%**	**2.03 [1.09; 3.78]**

Heterogeneity: $Tau^2 = 0.4683$; $Chi^2 = 60.92$, df = 4 ($P < 0.01$); $I^2 = 93\%$

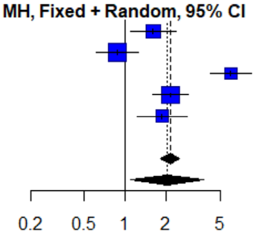

Supplementary Figure 2: Forest plot of comparisons for all polymorphisms associated with disease in the pooled studies with control groups in Hardy–Weinberg equilibrium.

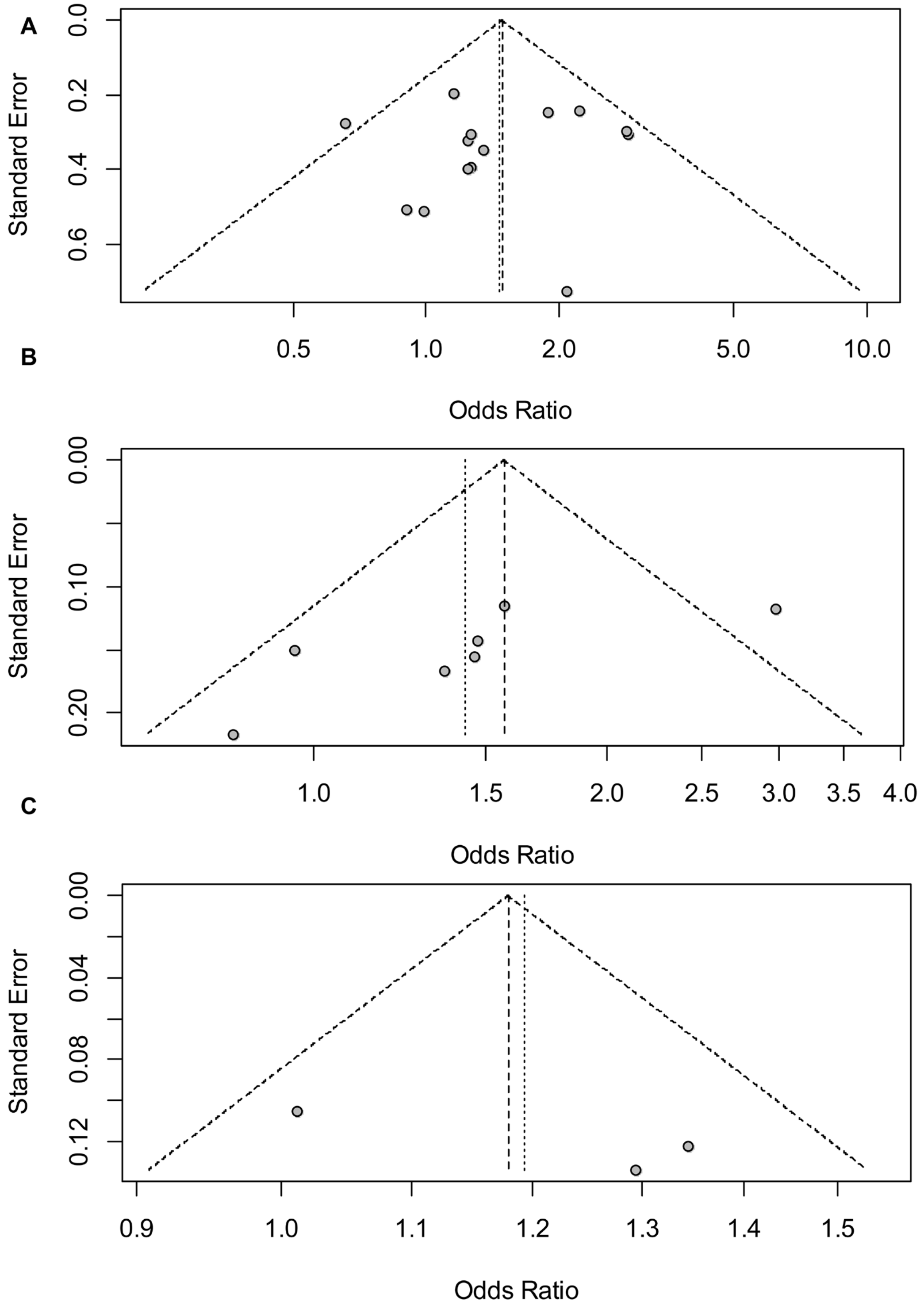

Supplementary Figure 3: Funnel plot of allele comparison of SNP rs11556218 and cancer (**A**), rs11556218 and CVD (**B**) and rs4778889 and gastric cancer (**C**).

Supplementary Table 1: Search strategy in selected databases

**Medline (PubMed MeSH terms)**
(((((“Polymorphism, Genetic”[Mesh]) OR “Genetic Techniques”[Mesh]) OR “Genotype”[Mesh]) OR “Alleles”[Mesh])) AND “Interleukin-16”[Mesh].
Free term: IL16 AND Polymorphism
**Scopus** (Article title, Abstract, Keywords)
“Polymorphism, Genetic” OR “Genetic Techniques” OR Genotype OR Alleles AND “Interleukin-16”
**Web of Science (Topic)**
(TS = (Polymorphism, Genetic OR Genetic Techniques OR Genotype OR Alleles) AND TS = Interleukin-16)

Supplementary Table 2: Covariables published in selected case-control studies of cardiovascular diseases

**Study (group)**	**Age (Mean ± SD)**	***P***	**Male (%)**	***P***	**Hypertension (%)**	***P***	**Diabetes (%)**	***P***	**Smoking (%)**	***P***
Liu 2013 (Case)	57.2 ± 11.8	0.11	122 (61.6)	0.38	119 (60.1)	**<0.01**	40 (20.2)	**<0.01**		
Liu 2013 (Control)	55.4 ± 11.7		155 (65.7)		22 (9.3)		8 (3.4)			
Tong 2013 (Case)	61.4 ± 8.7	0.26	243 (74.6)	**<0.01**	159 (48.8)	**<0.01**	136 (41.7)	**<0.01**	135 (41.4)	**<0.01**
Tong 2013 (Control)	60.6 ± 9.6		210 (61.6)		128 (37.5)		67 (19.6)		93 (27.3)	
Chen 2011 (1) (Case)	57.2 ± 7.8	**0.01**	243 (81.0)	**<0.01**	126 (42.0)	0.18	45 (15.0)	**<0.01**	150 (50.0)	**<0.01**
Chen 2011 (1) (Control)	59.1 ± 11.6		263 (66.2)		147 (37.0)		24 (6.0)		147 (37.0)	
Chen 2011 (2) (Case)	61.9 ± 10.9	0.07	340 (80.2)	**<0.01**	230 (54.2)	**<0.01**	94 (22.2)	**<0.01**	220 (51.9)	**<0.01**
Chen 2011 (2) (Control)	60.5 ± 10.4		227 (68.4)		107 (32.2)		33 (9.9)		81 (24.4)	
Wu 2011 (Case)	62.8 ± 11.6	0.30	95 (60.5)	0.76	50 (31.8)		26 (16.6)			
Wu 2011 (Control)	61.6 ± 10.4		119 (58.9)							

**Study (group)**	**Triglyceride (mmol/L)**	***P***	**Total Cholesterol (mmol/L)**	***P***	**HDL (mmol/L)**	***P***	**LDL (mmol/L)**	***P***
Tong 2013 (Case)	2 ± 1.1	**0.01**	4.2 ± 1.0	**<0.01**	1.2 ± 0.4	**<0.01**	2.5 ± 0.9	**<0.01**
Tong 2013 (Control)	1.8 ± 0.9		4.7 ± 1.0		1.5 ± 0.4		2.9 ± 0.9	
Chen 2011 (1) (Case)	2.1 ± 1.4	**<0.01**	4.1 ± 1.4	**<0.01**	1.1 ± 0.4	**<0.01**	2.5 ± 1.1	**<0.01**
Chen 2011 (1) (Control)	1.6 ± 1.0		4.8 ± 0.9		1.7 ± 0.4		2.9 ± 0.8	
Chen 2011 (2) (Case)	1.9 ± 1.1	0.99	4.1 ± 1.1	**<0.01**	1.2 ± 0.3	**<0.01**	2.5 ± 1.0	**<0.01**
Chen 2011 (2) (Control)	1.9 ± 1.3		4.7 ± 1.0		1.5 ± 0.4		2.8 ± 0.8	

Supplementary Table 3: General characteristics of selected *IL16* polymorphisms studies. See Supplementary Table 3
