# Supplementary material for: Association of functional IL16 polymorphisms with cancer and cardiovascular disease: a meta-analysis
Source: Oncotarget. 2020 Sep 8;11(36):3405–17. doi: 10.18632/oncotarget.27715 (PMC7486693; doi:10.18632/oncotarget.27715)
Supplement: Supplementary file 2 [file oncotarget-11-3405-s002.pdf]

Supplementary Table 3: General characteristics of selected *IL16* polymorphisms studies

Study	Year	Population	Disease	Group	n	rs4778889			HWEc	n	rs11556218			HWEc	n	rs4072111			HWEc	n	rs1131445			HWEc
						T/T	T/C	C/C			T/T	T/G	G/G			C/C	C/T	T/T			T/T	T/C	C/C	
Shih *et al.* [43]	2020	Taiwanese	Oral cancer	Case	958	633	293	32	0.99	958	633	269	56	0.76	958	588	332	38	0.87					
				Control	958	621	302	35		958	693	239	26		958	571	346	41						
Wu *et al.* [44]	2020	Taiwanese	Lung cancer	Case	358	234	113	11	0.97	358	201	131	26	0.71	358	224	117	17	0.98					
				Control	716	456	239	21		716	536	161	19		716	441	247	28						
He *et al*. [81]	2018	Chinese	Gastric cancer	Case	479	267	182	30	0.87	479	306	151	22	0.99	479	334	126	19	0.97	479	221	211	47	0.98
				Control	483	266	192	25		483	308	157	18		483	345	122	16		483	210	222	51	
Li *et al*. [82]	2018	Chinese	Papillary thyroid carcinoma	Case	538	323	190	25	0.91	538	242	252	44	0.63	538	329	186	23	0.63					
				Control	625	373	217	35		625	350	247	28		625	357	241	27						
Yang *et al*. [73]	2018	Chinese	Acute coronary syndrome	Case	238	102	107	29	0.45	238	133	71	34	**<0.01**										
				Control	178	71	75	32		178	96	46	36											
Kashfi *et al*. [67]	2016	Iranian	Gastric cancer	Case											300	246	52	2	0.76	300	156	119	25	0.66
				Control											256	184	65	7		256	111	121	24	
MaiMaiTiMin *et al*. [83]	2016	Chinese	Breast cancer	Case						230	118	67	45	0.05	230	106	95	29	0.99					
				Control						230	162	50	18		230	110	98	22						
Tang *et al*. [84]	2016	Chinese	Osteosarcoma	Case	358	215	127	16	0.98	358	165	174	19	0.77	358	218	124	16	0.77					
				Control	402	240	140	22		402	235	151	16		402	229	158	15						
Yang *et al*. [85]	2016	Chinese	Renal cell cancer	Case	273	132	113	28	0.82	274	149	110	15	0.63										
				Control	274	176	84	14		274	155	107	12											
Yao *et al*. [86]	2016	Chinese	Ovarian cancer	Case	220	113	62	45	0.08						220	101	90	29	0.98					
				Control	220	162	45	13							220	110	93	17						
Wang and Zhu [87]	2015	Chinese	Renal cell cancer	Case	181	82	77	22	0.85	181	94	75	12	0.86										
				Control	278	160	106	12		278	155	108	15											
Luo *et al*. [88]	2014	Chinese	Glioma	Case	216	142	68	6	0.85	216	90	119	7	0.32	216	138	66	12	0.85					
				Control	275	165	99	11		275	152	114	9		275	162	101	12						
Qin *et al*. [89]	2014	Chinese	Nasopharyngeal carcinoma	Case	75	39	36	0	0.11	75	32	37	6	0.84	75	41	34	0	0.05					
				Control	75	49	26	0		75	46	26	3		75	44	31	0						
Hai-Feng *et al*. [90]	2013	Chinese	Coronary heart disease	Case	261	139	89	33	0.06	260	175	64	21	**<0.01**						260	120	90	50	0.05
				Control	281	151	91	39		282	191	63	28							282	137	95	50	
Huang *et al*. [91]	2013	Chinese	Coronary artery disease	Case																651	299	295	57	0.98
				Control																428	186	196	46	

Supplementary Table 3: (continued)

Study	Year	Population	Disease	Group	n	rs4778889			HWEc	n	rs11556218			HWEc	n	rs4072111			HWEc	n	rs1131445			HWEc
						T/T	T/C	C/C			T/T	T/G	G/G			C/C	C/T	T/T			T/T	T/C	C/C	
Liu *et al*. [63]	2013	Chinese	Ischemic stroke	Case	198	124	66	8	0.85	198	88	102	8	0.52	198	125	63	10	0.89					
				Control	236	138	88	10		236	136	94	6		236	141	85	10						
Tong *et al*. [92]	2013	Chinese	Coronary heart disease	Case	326	170	106	50	0.13	326	223	69	34	0.72						325	157	112	56	0.08
				Control	341	201	107	33		340	248	80	12							341	171	116	54	
Zhang and Wang [93]	2013	Chinese	Gastric cancer	Case	347	188	114	45	0.47	347	165	114	68	**0.03**	347	242	56	49	**<0.01**	347	165	114	68	**0.02**
				Control	347	212	106	29		346	174	112	60		347	251	54	42		346	174	112	60	
Azimzadeh *et al*. [72]	2012	Iranian	Colorectal cancer	Case																249	103	110	36	0.93
				Control																394	201	159	34	
Azimzadeh *et al*. [94]	2011	Iranian	Colorectal cancer	Case	260	178	73	9	0.44	260	62	178	20	0.47	260	196	56	8	0.99					
				Control	405	274	112	19		405	124	226	55		405	324	77	4						
Chen *et al*. [95][a]	2011	Chinese	Coronary artery disease	Case	300	173	117	10	0.98	300	42	235	23	0.77										
				Control	397	241	138	18		397	232	149	16											
				Case						424	148	264	12	0.27										
				Control						332	178	144	10											
Li *et al*. [40]	2011	Chinese	Hepatocellular carcinoma	Case	206	158	42	6	0.83	206	122	62	22	0.13	206	110	80	16	0.89					
				Control	264	182	76	6		264	160	78	26		264	136	104	24						
Wu *et al*. [96]	2011	Chinese	Coronary artery disease	Case	157	95	55	7	0.91	157	61	92	4	0.48										
				Control	202	117	75	10		202	107	87	8											
Zhu *et al*. [97]	2010	Chinese	Renal cell cancer	Case	335	199	122	14	0.89															
				Control	340	171	135	34																
Gao *et al*. [98]	2009	Chinese	Nasopharyngeal carcinoma	Case	206	131	65	10	0.99	206	91	109	6	0.38	206	111	87	8	0.58					
				Control	373	228	128	17		373	210	151	12		373	221	139	13						
Gao *et al*. [68]	2009	Chinese	Colorectal and gastric cancer	Case	596	363	209	24	0.99	596	237	331	28	0.56	596	379	195	22	0.85					
				Control	480	294	164	22		480	265	197	18		480	283	179	18						

n: Case/control number of individuals, given by the sum of genotypes in the collected data.

[a] This study consisted of two independent groups of cases and controls.

HWEc: *P* value for Hardy-Weinberg equilibrium goodness-of-fit test in control groups (P< 0.05).
